# A Review of the Biology of Chikungunya Virus Highlighting the Development of Current Novel Therapeutic and Prevention Approaches

**DOI:** 10.3390/pathogens14101047

**Published:** 2025-10-16

**Authors:** Geovana Martelossi-Cebinelli, Jessica A. Carneiro, Kelly M. Yaekashi, Mariana M. Bertozzi, Beatriz H. S. Bianchini, Fernanda S. Rasquel-Oliveira, Camila Zanluca, Claudia N. Duarte dos Santos, Rachel Arredondo, Tiffani A. Blackburn, Rubia Casagrande, Waldiceu A. Verri

**Affiliations:** 1 Laboratory of Pain, Inflammation, Neuropathy and Cancer, Department of Immunology, Parasitology, and General Pathology, Londrina State University, Londrina 86057-970, Brazil; geovana.martelossi@uel.br (G.M.-C.); kelly.megumi.yaekashi@uel.br (K.M.Y.);; 2 Department of Pharmaceutical Sciences, Center of Health Science, Londrina State University, Londrina 86057-970, Brazil; 3 Vascular Biology Program, Department of Surgery, Boston Childrens’s Hospital-Harvard Medical School, Karp Research Building 1 Blackfan St, Boston, MA 02115, USArachel.arredondo@childrens.harvard.edu (R.A.);; 4 Laboratory of Molecular Virology, Carlos Chagas Institute/Fiocruz Paraná, Curitiba 81310-020, Brazil

**Keywords:** alphavirus, immunopathology, monoclonal antibodies, antivirals, vaccines

## Abstract

Chikungunya virus (CHIKV) is an arthritogenic alphavirus transmitted primarily via *Aedes aegypti* and *Aedes albopictus* mosquitoes. Since its identification, CHIKV remained confined to parts of Africa and Asia until the early 2000s, when it expanded to other continents, causing epidemics. Structurally, it is an enveloped virus with a positive-single-stranded RNA genome, which encodes four non-structural proteins (nsP1-nsP4), responsible for viral replication, and five structural proteins (C, E3, E2, 6K, and E1), which form the capsid and envelope. Of these proteins, glycoproteins E1 and E2 are essential for cell recognition and membrane fusion, determining infectivity and viral tropism. CHIKV replication occurs in the cytosol of different cell types, triggering an intense inflammatory and immune response, which manifests clinically as Chikungunya fever (CHIKF). Despite its epidemiological impact, current treatment is limited to symptomatic approaches, including the use of analgesics and anti-inflammatories, as no specific antiviral therapies are available. In response, promising advances are being made, including the development of vaccines, targeted antivirals, and immunotherapies. This article aims to review the main aspects of viral biology, epidemiology, and immunopathogenesis of CHIKV infection, in addition to discussing the main advances in the development of new therapeutic approaches for its control.

## 1. Introduction

Chikungunya virus (CHIKV) is an arthropod-borne virus (arbovirus) belonging to the *Alphavirus* genus and *Togaviridae* family, transmitted primarily by *Aedes (Ae.) aegypti* and *Ae. albopictus* mosquitoes. First identified in Tanzania in 1952, CHIKV has since emerged as a significant global public health concern, particularly in tropical and subtropical regions [1]. Clinically, CHIKV infection manifests with fever, hence it commonly being referred to as Chikungunya fever (CHIKF). However, there are other characteristics such as acute onset of high fever, rash, and debilitating polyarthralgia, which can persist for weeks or months [2]. In some cases, the disease progresses to a chronic phase, leading to long-term joint pain and disability, severely impacting patients’ quality of life [3]. Therefore, we will use the term CHIKV disease to reflect that fever is not the only symptom.

Over the past few decades, the incidence of CHIKV infections has increased dramatically, with major outbreaks reported across Africa, Asia, the Indian subcontinent, and the Americas [4]. The re-emergence and rapid geographic expansion of the virus highlight its growing importance in both epidemiological surveillance and clinical research [5]. From both scientific and clinical perspectives, CHIKV poses several significant challenges, such as the scarcity of effective treatments, unavailability of an approved vaccine (until recently), and the insufficient knowledge regarding its disease mechanisms and long-term effects [6].

This article aims to provide a comprehensive overview of CHIKV, focusing on its virological characteristics, epidemiology, immunopathogenesis, and the current state of research in the search for new therapeutic approaches. By highlighting the scientific and clinical relevance of CHIKV disease, the objective is to support further efforts in prevention, diagnosis, and treatment strategies.

## 2. General Aspects of Chikungunya

### 2.1. Epidemiology of Chikungunya Virus

**Figure 1:**
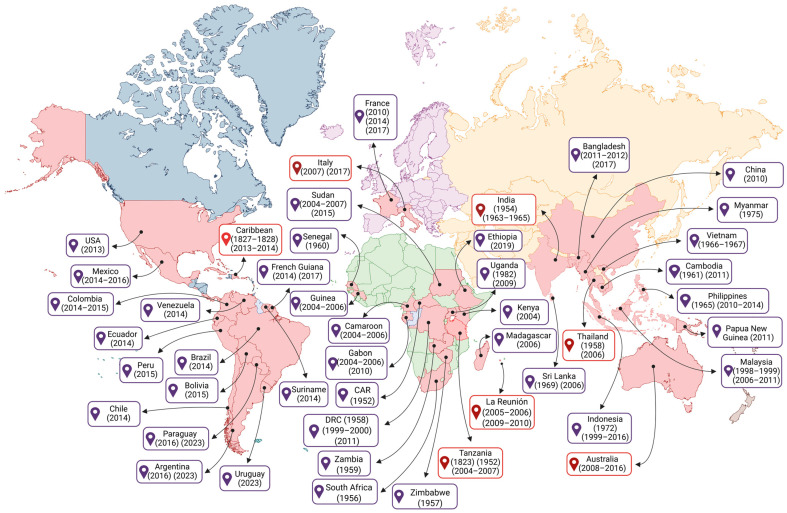
Global epidemiology of the Chikungunya virus over time. Initially reported on the African continent, CHIKV, driven by trade and the traffic of travelers between different regions of the world, has spread to every continent except Antarctica. That figure presents a chronological sequence of CHIKV amplification around the world over the years, considering the year in which it was first reported or in which new outbreaks or epidemics occurred. The red text boxes highlight the countries where the first reported cases of the disease occurred on the continent. Each continent is represented by a color: blue (Americas), green (Africa), purple (Europe), orange (Asia), and brown (Oceania). Countries where CHIKV presence has been reported are shown in red. Please note that not all affected countries are shown. Created in BioRender. Oliveira, F. (2025) https://BioRender.com/15wrcvb (accessed on 12 October 2025).

The first reported epidemiological cases of fever, arthritis, and rashes resembling the CHIKF include cases in Zanzibar (Africa) in 1823, and an epidemic on the island of Saint Thomas (Caribbean) in 1827 and 1828 [7]. Some authors propose that the spread of the CHIKV beyond African territory may have begun in the mid-18th century when sailing ships carried humans and infected *Ae. aegypti* mosquitoes in sufficient numbers for the virus to circulate on board the ships, where the water stored for the crew was conducive to the reproduction and propagation of mosquitoes [8]. Since then, CHIKV has reached various territories around the world, resulting in outbreaks, endemics, and epidemics (Figure 1).

However, it was not until 1952 that the CHIKV was isolated from the serum of a febrile patient during an outbreak on the Makonde plateau, located in south-eastern Tanzania in East Africa; and in 1953, it was isolated for the first time from *Ae. aegypti* mosquitoes, one of the main vectors of this virus [9]. Based on the disabling and debilitating symptoms presented by patients with severe arthralgia, the disease was given the name “Chikungunya” [10].

After the initial isolation of CHIKV, the first evidence of clinical infection was reported in an Indian patient in 1954 [11,12,13], as well as outbreaks in other African countries such as South Africa (1956), Zimbabwe (1957), the Democratic Republic of Congo (1958), Zambia (1959), Senegal (1960), Central African Republic (1982), and Uganda (1982) [14]. On the Asian continent, the first laboratory-confirmed outbreak occurred in Bangkok, Thailand (1958) [12]. In the following years, cases were recorded in other Asian countries, such as Cambodia (1961) [15], India (1963–1965, 1973) [16], the Philippines (1965), Vietnam (1966–1967), Sri Lanka (1969), Indonesia (1972), and Myanmar (1975) [12,17,18,19,20,21,22]. In the 2000s, new outbreaks of CHIKV were reported form several countries across the African continent, including the Kenya (2004), Cameroon (2006), Democratic Republic of Congo (1999–2000, 2011), Gabon (2006, 2007 and 2010), Madagascar (2006), Senegal (2009), Ethiopia (2019), and Sudan (2015) [23,24].

On the Asian continent, outbreaks of CHIKV disease were reported during 1998–1999 and 2001–2003 in Malaysia and Indonesia, respectively [25]. Following these outbreaks, CHIKV spread to the Indian Ocean islands, including Comoros, La Réunion, Mauritius, and Mayotte [26], and subsequently, infected air travelers from these epidemic regions reached Europe, Asia, and the Americas, contributing to the spread of CHIKV [8]. Between 2005 and 2006, more than 300,000 cases of CHIKV infection were reported on La Réunion Island [26], located in the Indian Ocean and belonging to France. Years later, between 2009 and 2010, the number of cases of CHIKV infection in La Réunion increased again [27]. During the outbreak in the Indian Ocean islands and Asia, cases of CHIKV were also reported in Europe and the Americas. Thus, since 2005, CHIKV transmission has reached global levels, with outbreaks in Asia, including Indonesia (1999–2016), Sri Lanka (2006), Malaysia (2006–2011), Thailand (1965, 2010–2014), Singapore (1965, 2006–2011), China (2010), Cambodia (2011), Bangladesh (2011–2012, 2017), and India (2005–2018) [28]. Moreover, in March 2025, La Réunion reported a new wave of outbreaks, with over 13,000 CHIKV cases in all the municipalities on the island. Due to the exponential number of cases and the increase in outbreaks, this led to the activation of level 4 of the OSERC “Arbovirus” system, corresponding to the circulation of an epidemic of medium intensity [29]. In addition, since the CHIKV outbreak in La Réunion in 2005, cases of infection have become increasingly frequent in Europe, with cases in Italy (2007, 2017), appearing to have originated from the introduction of the virus by a traveler returning from India, resulting in more than 200 cases of local transmission. Subsequently, there were new autochthonous cases in Lazio, in central Italy [24]. Later, another similar outbreak occurred in the southern region of France (2010, 2014, and 2017), which seems to have originated from the introduction of one of the CHIKV vectors, the *Ae. albopictus* mosquitoes [24,30].

In Oceania, the first reported cases of CHIKV infection were in 2011 related to an autochthonous transmission of CHIKV in New Caledonia, a French territory located in the Pacific Ocean, in the Melanesian region, characterizing the first time the virus was detected in the Pacific [31]. The following year, another outbreak was recorded in Papua New Guinea, also located in the Melanesian region of Oceania [32]. In 2013, outbreaks occurred in other Pacific regions, such as New Caledonia, Tonga, American Samoa, and the Independent State of Samoa. In 2015, there were also outbreaks in the Cook Islands [11]. In Australia, the first cases of CHIKV infections were reported in 2008, mainly from travelers infected in other countries [33]. Pyke et al. (2018) showed that CHIKV cases between 2010 and 2016 were imported into Australia by patients traveling from Southeast Asia, the Pacific, and the Americas [34].

CHIKV was reported as the cause of three epidemics in the Americas, with 671,268–1,089,982 cases reported per year between 2014 and 2016, and more than 97,000 cases per year from 2017 to 2023 [35]. The Asian lineage was introduced to the island of Saint Martin in the United States of America (USA) in October 2013 and from there spread through the Caribbean, Central America, northern South America, and North America, infecting almost 1,400,000 people [36,37]. In 2014, one of the largest CHIKV epidemics in the Americas occurred in the Latin Caribbean (Cuba, the Dominican Republic, Puerto Rico, Haiti, Guadeloupe, and Martinique), followed by the Non-Latin Caribbean region (Jamaica, the Bahamas, the US Virgin Islands, and Aruba). Later, the Central American and Andean regions were also affected [35]. In 2014, CHIKV arrived in the USA with local transmission occurring in Florida, Texas, Puerto Rico, and the USA Virgin Islands [38].

In South America, cases of CHIKV infection have been reported in all countries, this includes Argentina (2015), Bolivia (2015), Brazil (2014), Chile (2014), Colombia (2014–2015), Ecuador (2014), French Guiana (2014, 2017), Paraguay (2016, 2023), Peru (2015), Suriname (2023), Uruguay (2023), and Venezuela (2014) (PAHO/WHO). Among those countries, Brazil is considered the epicenter of the CHIKV epidemic in South America. Brazil is the largest and most populous country in Latin America, making it particularly susceptible to the CHIKV alphavirus. In addition, the country’s climate is suitable for the *Ae.* vector. In August 2014, local transmission of the Asian American sublineage of CHIKV was reported in the city of Oiapoque in the state of Amapá, and the new East Central South African (ECSA) sublineage (ECSA American) was detected in the city of Feira de Santana, in the state of Bahia [39]. The Asian American sublineage appears to have been introduced via French Guiana, which borders Brazil through the state of Amapá, and this sublineage appears to be restricted to two of the seven states that make up the Northern region (Amapá and Roraima) [39,40]. In subsequent years, the ECSA strain spread to other Brazilian northeastern states [40]. In 2016, the first detection of *Ae. aegypti* naturally infected with the ECSA genotype was reported, supporting the hypothesis that this species was acting as the main vector of CHIKV outbreaks [41]. In recent years, the ECSA American sublineage has become predominant in all Brazilian regions, as well as expanding into countries such as Haiti, Argentina, Uruguay, and Paraguay [42]. Furthermore, while the Asian American sublineage of CHIKV has not been reported since 2018 in the Americas, the ECSA American sublineage continues to cause outbreaks in Brazil, Uruguay, Paraguay, and Argentina [35]. By the year 2023, CHIKV cases in North America represent 0.3% of all reported cases in the Americas (12,172 out of 3,684,554), with most cases occurring in Mexico (12,034, equivalent to 92.7% of cases) [35]. In 2024, the American continent reported 1,008,430 cases, with the highest number occurring in North America, specifically in the USA (345,426 cases) (PAHO/WHO).

From January to May 2025, 220,000 cases and 80 deaths were reported in America, Africa, Asia, and the islands of the Indian Ocean, with a predominance of cases in South America [29]. Then, at the end of June and beginning of July 2025, 14 autochthonous cases were reported in France [43]. The continuing occurrence of CHIKV cases indicated that efforts to control and treat CHIKV disease was an ongoing major public health problem, especially for populations in tropical and subtropical countries, where the climate, fauna, and flora are conducive to vector survival and viral transmission.

### 2.2. Main Regional Genotypes of Chikungunya Virus over Time

**Figure 2:**
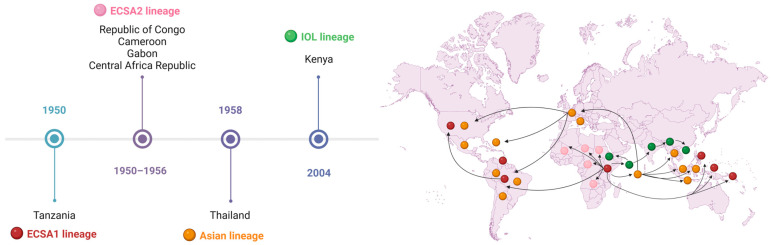
The chronology of the emergence of the different *Alphavirus chikungunya*’s strains over time, following their geographical origin and subsequent spread. CHIKV has four main genotypes, resulting from adaptive mutations that have occurred over time. Chronologically, the first genotype described refers to the East South Central African lineage (ECSA1) (in red), followed by the West African lineage (ECSA2) (in pink), the Asian lineage (in yellow), and the Indian Ocean lineage (IOL) (in green), which spread to various countries around the world. Created in BioRender. Oliveira, F. (2025) https://BioRender.com/15wrcvb (accessed on 28 February 2025).

The CHIKV has four distinct genotypes, recognized and classified according to the regions in which the virus has been detected or a genotypic adaptation has been recognized (Figure 2). The four main genotypes are the ECSA lineage, the West African lineage (also referred to as ECSA2), the Asian lineage, and the Indian Ocean Lineage (IOL) [34].

This geographical expansion of the different genotypes was facilitated by the intense circulation of infected people between countries. In particular, the ECSA lineage can be subdivided into ECSA1 (referring to the virus that infected the human population in Tanzania in 1950) and ECSA2 (which contains viral sequences obtained from the subsequent outbreaks that affected the Republic of Congo, Cameroon, Gabon, and the Central African Republic) [44].

Evolutionary studies have shown that the West African genotype evolved into a distinct variant when it spread to Asia, called the Asian genotype [45]. In 2004, during an outbreak in Lamu and Mombasa on the Kenyan coast, a new strain of ECSA was registered, which would later be classified as IOL, which circulated throughout the Indian Ocean region in 2005 and was introduced into India in 2006 [28], infecting around 2,000,000 people.

Tsetsarkin et al. (2007) carried out a more in-depth analysis of the microevolution of the CHIKV genome obtained during the outbreak on La Réunion Island in the Indian Ocean, identifying a mutation from alanine to valine at position 226 (E1-A226V) in the E1 glycoprotein (a viral envelope glycoprotein essential for virus–host cell fusion) [46]. This residue alteration conferred greater infectivity of the virus on the vector *Ae. albopictus*, amplifying the capacity for viral replication in the vector’s cells and dissemination to secondary organs, which, consequently, caused a slight increase in the transmission of the virus by *Ae. albopictus*, favoring it as the main vector in the region [46,47,48]. This mutation seems to have contributed to the viral spread in the region and the significant increase in case numbers. The CHIKV E1-226V variant has also been implicated in outbreaks occurring in Africa (Cameroon, Gabon, and Congo) [49,50,51], Europe (Italy) [52], and Southeast Asian regions (Malaysia, Singapore, Thailand, China, Cambodia, and Bhutan) [15,53,54,55,56].

Other adaptive mutations affecting the E2 gene (encoding the E2 glycoprotein, a surface protein crucial for virus infection and pathogenicity) have also been reported, resulting in increased transmission by the *Ae. albopictus* vector. These E2 protein adaptive mutations occurred on the Asian continent. The first to be described was E2-K252Q, which occurred in the state of Kerala (India) in 2007 and spread throughout Southeast Asia, where it was isolated from in 2008 [15,57,58]. In 2008, the adaptive mutations E2-V222I and E1-K211N were reported in Sri Lanka [59]. In 2009–2010, the adaptive mutation E2-L210Q was detected in India. All these mutations are related to the increased infection and transmission of CHIKV through *Ae. albopictus* mosquitoes [60].

Moreover, the different genotypes exhibit differences in their transmission cycles (Topic 2.3). While the Asian genotype seems to be transmitted mainly in an urban cycle with *Ae. aegypti* and *Ae. albopictus* mosquitoes, the African genotype seems to be more related to a sylvatic cycle, whose main vectors are the *Ae. furcifer* and *Ae. africanus* mosquitoes [61]. Furthermore, other adaptive mutations have been demonstrated that favor the transmission of the virus through the *Ae. albopictus* vector, such as T98A (E1) [62], L210Q (E2) [63], K252Q (E2) [63], I211T (E2) [64] and GD60D (E2) [64].

In particular, the genomes of the ECSA strain of CHIKV circulating in Brazil do not have the adaptive changes E1-A226 or E2-L210Q of *Ae. albopictus*, but have other mutations such as E1-K211T, E1-N335D, E1-A377V, E1-M407L, and E2-A103T, which seem to improve viral transmission in the *Ae. aegypti* vector, but not for *Ae. albopictus* [65]. These mutations significantly increased the infectivity of the virus (13×), its dissemination (15×), and transmission (62×) [65]. In South America, most of the CHIKV genomes available and used in research were obtained from samples collected between 2014 and 2015, the peak of the epidemic in the Americas, and, more recently, in samples obtained during recent epidemics in Brazil and Paraguay between the years 2021 and 2023. To date, 62.6% of all CHIKV genomes shared on NCBI GenBank are from Brazil, and 99.4% of them belong to the ECSA American sublineage [35].

Some factors have been identified as major influencers of the spread of CHIKV, such as the increase in air travel, allowing the flow of infected people between regions, the lack of prior exposure of human populations in the Indian Ocean basin and South Asia, the expansion of mosquito vectors such as *Ae. albopictus* into non-native regions (from native Asia to the islands of the Indian Ocean basin, Africa, and southern Europe), which has been facilitated by global trade, as well as the various adaptive mutations that the new strains of the virus have undergone over the years, conferring greater transmissibility and virulence [46,47,60,66].

Hence, monitoring these adaptive CHIKV mutations that occur during outbreaks and epidemics is essential. Since these genetic alterations can enhance viral transmission and survival. Thus, mapping and identifying these mutations could be crucial for controlling potential outbreaks and for developing new pharmacological therapies that include these alterations in viral structures.

### 2.3. Vectors of Chikungunya Virus

*Ae. aegypti* originated in sub-Saharan Africa and was first identified as an arbovirus vector in Cuba in 1900 [67]. This vector measures 4–7 cm and has a dark coloration (black or brown) with white or silver stripes arranged on the body and legs. In addition to having a high potential for pathogenic transmission to humans due to its purely anthropophilic habits and reproduction that target domestic (urban) and peridomestic environments [68,69,70,71]. This vector is more ecologically flexible compared to other vectors, *Ae. albopictus*, for example, because its geographic distribution is wider, especially in tropical and subtropical environments since this vector can be found in suburban and rural habitats. Furthermore, despite feeding mainly on humans, this vector can also infect a wide range of hosts, such as livestock, amphibians, reptiles, and birds, with different types of viruses [72,73].

First described in the Indian city of Calcutta, *Ae. albopictus*, also known as the “Asian tiger mosquito”, is native to Southeast Asia, the Western Pacific islands, and the Indian Ocean [74], and is frequently found in areas of extensive vegetation cover and more dispersed human populations; however, it has also been described in transitional environments with relatively low vegetation cover and generally coexisting with *Ae. aegypti* [75,76,77,78,79]. This mosquito has a black-and-white coloration, white bands on the legs, a median longitudinal band of silvery scales on the mesonotum, and a scaleless clypeus [80]. However, this species has spread throughout the tropics, mainly due to the development of human trade [81]. Despite being an opportunistic and zoophilic mosquito, when allowed to choose, this *Aedes* species shows a preference for feeding on human blood compared to the blood of other animals [82]. Furthermore, the results of some studies demonstrated that CHIKV was transmitted vertically from infected female *Ae. albopictus* to their offspring [83,84,85,86].

*Ae. aegypti* and *Ae. albopictus* use artificial container habitats for breeding and laying eggs so they can withstand dry conditions, ensuring survival even during unfavorable times, such as less rainy seasons, which facilitates the geographic expansion of these vectors [87,88,89,90]. While *Ae. aegypti* is restricted to warmer climates due to its inability to diapause, *Ae. albopictus* can be found in cooler and temperate regions because its eggs can enter diapause and overwinter—though the adults themselves can’t survive winter. [91,92]. The main breeding habitat of *Ae. aegypti* and *Ae. albopictus* is freshwater and their eggs are resistant to desiccation and high humidity, allowing them to hatch when conditions become favorable, giving rise to larvae [93]. The extensive geographic distribution of *Ae. albopictus*, jointly with mutations that improve the fitness and infectivity of CHIKV in this vector, may contribute to the expansion of the virus to temperate ecosystems in other regions, as observed by small outbreaks in France and Italy [52,94]. In addition, temperature changes are also an important influence of the vectorial capacity and transmission of CHIKV [92,95,96]. The increase in global temperature may also influence the migration of vectors restricted to warmer climates, such as *Ae. aegypti*, facilitating the spread of arboviruses, as seen in Europe, where the first autochthonous cases of flavivirus infections were reported [97,98,99].

Another factor that influences the transmission of CHIKV is the microbiota of the digestive tract of the mosquito vector. It has been described, for example, that the presence of the intracellular bacterium *Wolbachia* (*Wb*), which infects *Ae. mosquitoes*, interferes with the mosquito’s immune response, resulting in increased expression of cellular factors such as thioester-containing proteins (TEPs), C-type lectins, defensins, diptericin, glycosaminoglycan-binding protein B1 (GNBPB1), serine protease Z1A (PZ1A), cactus, and cecropin, which help neutralize CHIKV proliferation within the mosquito [100,101,102].

### 2.4. Transmission of Chikungunya Virus

**Figure 3:**
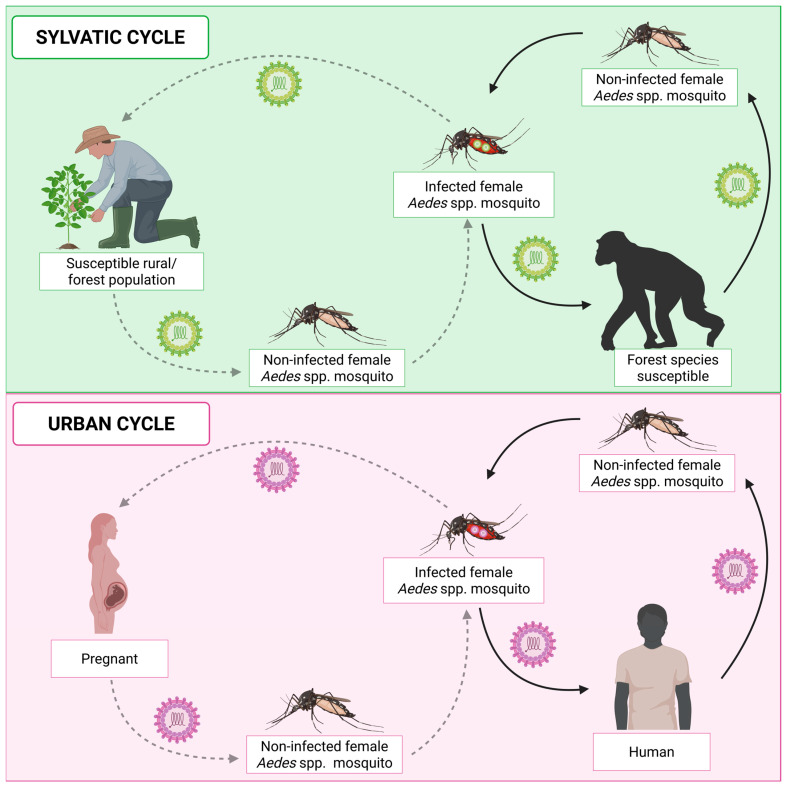
Chikungunya virus transmission cycles. CHIKV transmission can occur through the sylvatic cycle or the urban cycle. The sylvatic cycle occurs in forest regions where infected female *Aedes* mosquitoes transmit the virus to non-human primates (NHPs). As a result, uninfected female *Aedes* mosquitoes, when feeding on infected NHPs, become infected and perpetuate the cycle. In addition, humans who live in or frequent forest regions can also participate in this cycle, as accidental hosts of the virus. The urban cycle occurs in regions densely populated by humans and involves the transmission of the virus mainly by *Ae. aegypti* and *Ae. albopictus* mosquitoes to humans. In the urban cycle, vertical transmission from mother to child can also occur. In this type of transmission, the infected mosquito transmits the virus to a pregnant woman. In turn, the virus can be transmitted to the fetus or baby during the breastfeeding period. Created in BioRender. Oliveira, F. (2025) https://BioRender.com/15wrcvb (accessed on 10 August 2025).

CHIKV is transmitted by a sylvatic cycle (also known as enzootic) and an urban cycle (Figure 3). Transmission occurs mainly through the mosquito vectors *Ae. albopictus* and *Ae. aegypti*. However, CHIKV has also been detected in other species of the *Ae.* genus, such as *Ae. fucifer*, *Ae. taylori*, *Ae. luteocephalus*, *Ae. africanus*, *Ae. neoafricanus* and *Ae. cordellieri* [103]. The sylvatic cycle was demonstrated in the 1960s, in a study that isolated CHIKV from a pool of the forest mosquito *Ae. africanus* collected in the forest canopies of Uganda [104]. In this study, after infection of mice and *rhesus* monkeys with the virus, both species developed the disease, demonstrating that they are viable hosts for viral replication and propagation. Subsequently, another study using experimentally infected *vervet* monkeys (*Chlorocebus pygerythrus)* demonstrated that the animals produced CHIKV antibodies after infection, suggesting once again the involvement of non-human primates (NHP) in a sylvatic transmission cycle [105]. Thus, this cycle involves the transmission of the virus by forest-dwelling mosquito vectors to NHP (e.g., monkeys, baboons, and squirrels) [103]. Occasionally, humans may be accidentally infected when they move around or live near forests. In the urban cycle, the virus is transmitted by mosquito vectors to humans. CHIKV transmission depends on environmental factors, such as rainfall and altitude, ecological factors, including competent mosquito vectors, and social factors, including human mobility, socioeconomic status, and lifestyle [106,107,108,109].

CHIKV is transmitted primarily through the saliva of infected mosquitoes. When the female mosquito feeds on an infected person, the vector ingests the virus, which infects various tissues, such as the salivary glands. Subsequently, when the infected mosquito feeds on a naive (uninfected) individual, CHIKV is transmitted directly into the bloodstream or dermal tissue [4]. At this point, the components present in the saliva of the mosquito will be able to inhibit the interferon (IFN) signaling pathway, facilitating viral replication [110,111,112,113]. In the dermis, CHIKV can invade and replicate in dendritic cells (DCs) [114], macrophages [115], endothelial cells, epithelial cells [116], keratinocytes [117], melanocytes [118] and, above all, fibroblasts, which function as reservoirs for viral amplification [112,116,119,120,121]. CHIKV, like most alphaviruses, enters host cells through endocytosis mediated by the clathrin protein [122]. Moreover, the entry of CHIKV into the bloodstream seems to occur through viral binding, infection, or absorption by antigen-presenting cells, such as DCs and macrophages, which migrate to the lymphatic vessels and subsequently reach the bloodstream [123]. It is hypothesized that viral replication in the spleen, liver, endothelial cells, and monocytes contributes significantly to the high viremia typical of the acute phase [115,124,125,126]. From the blood, CHIKV can spread systemically and infect different tissues, such as those related to the symptoms of the disease: connective tissue, muscles and joints [115,119,121,127,128,129].

The first report of vertical transmission (mother to child) occurred during the La Réunion epidemic in 2005. On this occasion, the vertical transmission rate was close to 50% in mothers with high viremia during the intrapartum period [130]. Transmission from mother to fetus appears to occur through microtransfusions in the placental barrier or through the breakdown of the syncytiotrophoblast during uterine contractions [131,132]. In this context, the placenta seems to play a key role in this type of transmission, although it is not fully understood. CHIKV antigens have been detected in decidual cells, trophoblastic cells, endothelial cells, Hoffbauer cells and within the fetal capillaries of the placenta [133,134,135].

Chen et al. (2010) [136] evaluated CHIKV infection in pregnant *Rhesus* monkeys (*Macaca mulatta*, 7 and 15 years old and gestational days of 121–132 days) to evaluate the pathogenesis and the potential for transplacental transmission. The study demonstrated that viremia peaked 2–3 days after inoculation, with the development of intermittent fever (39.7 °C), increased joint temperature (1.19–9.4 °C) and circumference, appearance of erythematous skin rashes, severe leg swelling and high levels of inflammatory cytokines [interleukin (IL)-2, IL-6, IL-15, interleukin-1 receptor antagonist (IL1Ra), monocyte chemotactic protein-1 (MCP-1), and vascular endothelial growth factor (VEGF)]. In addition, through necropsy, viral ribonucleic acid (RNA) was observed in maternal lymphoid tissues associated with joints and the spinal cord. On the other hand, no viral RNA was detected in the germinal center in fetuses, which indicated the absence of transplacental transmission. This finding contrasts with studies conducted in humans, which demonstrate vertical transmission of CHIKV [119,137].

Moreover, it has been observed that postponing normal delivery or performing a cesarean section does not prevent CHIKV transmission from mother to fetus. Maternal infection results in obstetric complications such as miscarriage, pre-eclampsia, post-partum hemorrhage, premature birth, intrauterine death, oligohydramnios, and sepsis [138,139]. Infected neonates may present symptoms such as fever, refusal to breastfeed, skin rashes, skin hyperpigmentation, thrombocytopenia, and neurological complications, such as meningoencephalitis, cerebral edema, postnatal microcephaly and neurodevelopmental delay [140,141,142,143,144,145,146].

## 3. Virology and Structure of Chikungunya Virus

The term “togavirus” originated as informal jargon during early studies of arboviruses, particularly those isolated in the context of yellow fever research. The etymology is derived from the Latin toga, a Roman “mantle,” “cloak,” or “covering,” in reference to possessing a viral envelope [147]. Initially, the *Togaviridae* family included two genera: *Alphavirus* and *Flavivirus*. However, due to subsequent taxonomic revisions, flaviviruses were reclassified into their own family, *Flaviviridae*, based on distinct genomic and structural characteristics. As a result, the *Togaviridae* family now consists of a single genus, *Alphavirus*. All alphaviruses share a spherical virion architecture ~70 nm in diameter, and this genus includes CHIKV and other medically important viruses such as Sindbis virus (SINV), Semliki Forest virus, and Eastern equine encephalitis virus (EEEV) [147,148].

**Figure 4:**
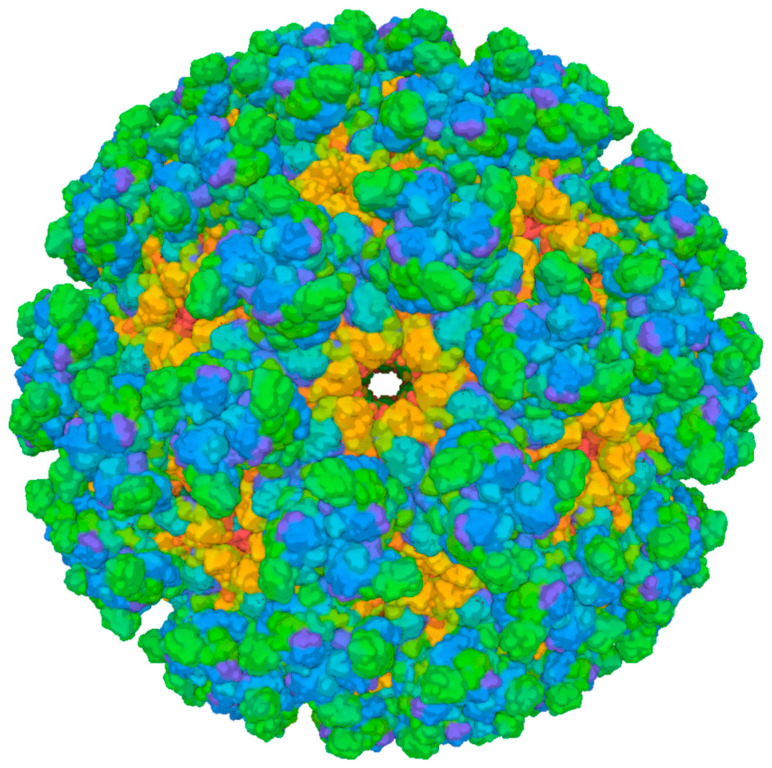
Cryo-EM structure of Chikungunya virus strain Senegal 37997 VLP (PDB 6NK5). Surface representation of the virus-like particle derived from electron cryo-microscopy. The different colors represent different protein chains. Green represents the E2 protein complex, blue represents the E1 protein complex, and orange represents the Capsid protein complex. Image from the RCSB PDB (RCSB.org) of PDB ID 6NK5 [182]. Created in BioRender. Oliveira, F. (2025) https://BioRender.com/15wrcvb (accessed on 17 August 2025).

The CHIKV shares characteristics with *Alphaviruses* and consists of spherical particles approximately 70 nm in diameter and exhibits icosahedral symmetry with triangulation [147,149,150]. CHIKV genome consists of a single-strand positive-sense RNA molecule approximately 12 kb in length, featuring a 5′ capped structure and a 3′ polyadenylated (poly(A)) tail. The CHIKV genome is separated into two open reading frames (ORFs). The 5′ ORF encodes four non-structural proteins (nsP; nsP1–4) required for viral replication and the 3′ ORF encodes five structural proteins (Capsid, E1, E2, E3, and a 6K protein) (Figure 4) [149,151,152,153].

NsP are essential for virus replication, protein modification, and immune evasion. The nsPs are mainly produced as a single polyprotein with distinct roles in viral genome replication, also known as replicase complex [154,155]. NsP1 caps the 5′ end of the new viral RNA independently of the host–cell capping machinery. It is the only nsP reported to bind membranes, and its membrane affinity is enhanced by, but not dependent on, a palmitoylation site [156,157,158]. The nsP2 has RNA helicase and RNA triphosphatase (RTPase) activity in its N-terminal domain, and its C-terminus harbors a cysteine protease domain, which cleaves the polyprotein into individual nsPs [159,160]. NsP3 has adenosine diphosphate (ADP)-ribosyl hydrolase activity and interacts with several host–cell proteins [161,162,163]. NsP4 is the RNA-dependent RNA polymerase directly responsible for producing new viral RNA [164,165].

CHIKV structural proteins form the virion and are translated from subgenomic viral RNAs. Briefly, the 3′ ORF is transcribed into a subgenomic positive-stranded RNA, which encodes five structural proteins after subsequent cleavage and maturation steps. It is also expressed as a polyprotein processed by viral and cellular proteases [166,167,168].

Three main structural proteins are expressed: capsid protein (CP), E1, and E2 viral glycoproteins. Two supplementary small structural proteins, E3 and 6K, and its translational frameshift product, transferase (TF), are also synthesized. These act as stabilization and regulation functions involved in viral glycoprotein assembly and particle budding [167,169,170,171,172].

The structure of the CHIKV virion consists of a single-strand RNA encapsulated by 240 copies of the highly basic CP. This protein assembles into an icosahedral nucleocapsid exhibiting T = 4 symmetry, a defining characteristic of alphaviruses [167,173]. Beyond its structural role, the CP mediates the genomic encapsulation within the nucleocapsid and actively engages in the viral budding process through interactions with the cytoplasmic domain of E2 glycoprotein and finally the virion assembly [167,172,174,175,176,177].

This nucleocapsid is enveloped by a host-derived lipid bilayer that contains 80 trimeric glycoprotein spikes. Each spike consists of three E1-E2 heterodimers; the primary function of the E1 subunit is to mediate membrane fusion during viral entry, which facilitates the release of the viral genome into the host cell cytoplasm [178]. The E2 subunit is critical for receptor recognition and binding to the host cell surface [174,179]. Additionally, the E2 subunit plays a crucial role in viral entry by initiating clathrin-dependent endocytosis, which allows for the internalization of the virion into the host cell [180,181].

The E3 glycoprotein acts as a chaperone, properly folding the E2 glycoprotein and inhibiting premature conformational changes in the E2–E1 heterodimer through the acidic secretory pathway [167,182]. E3 and E2 are initially synthesized as the precursor p62 protein, which is cleaved by furin during maturation [183,184]. While E3 is typically cleaved in mature virions, it can remain bound to the E2–E1 heterodimer in some alphaviruses, such as SINV and CHIKV [173]. The dissociation of E3 also depends on factors like the culture medium’s pH and infected cells’ confluency [185,186].

The 6K protein, despite its small size, is essential for virion assembly and release [187]. It shares an N-terminus with the TF protein, but their C-termini differ due to ribosomal frameshifting [188]. Both 6K and TF are hypothesized to form ion channels and are found in low amounts in virion particles [189]. They contribute to viral budding [189] and pathogenesis, but their exact roles in glycoprotein processing, assembly, budding, and particle stability remain unclear [189,190]. Additionally, their hydrophobic nature has excluded them from recombinant protein preparations used for structural studies, further limiting understanding of their precise functions [167,191].

### 3.1. Membrane Fusion and Entry to Host Cells

Viral entry into host cells comprises a sequence of well-defined stages: attachment, receptor binding, endocytosis, and membrane fusion. The process initiates with attachment, wherein viral particles engage with the cellular surface through interactions with attachment factors, which results in the concentration of viral particles without necessarily inducing conformational changes in the viral envelope proteins. Subsequently, receptor binding occurs, characterized by specific interactions between viral proteins and host cell receptors that can provoke structural rearrangements essential for entry. The virus is then internalized via endocytosis, often facilitated by pathways dependent on clathrin or caveolin, culminating in the release of the viral genome into the cytoplasm and the initiation of infection [151,181,192,193].

#### 3.1.1. Structural Components of Chikungunya Virus Involved in Entry

The entry of CHIKV into host cells is mediated by the structural proteins E1 and E2, which form heterodimers on the viral surface. These glycoproteins play essential roles in receptor binding and membrane fusion. Specifically, E2 is primarily responsible for the attachment to host cell receptors, while E1 facilitates the low-pH-dependent fusion process. During the viral assembly, the stability of the E1-E2 heterodimer is enhanced by the presence of the E3 glycoprotein, which prevents premature fusion [151,193].

E1 glycoprotein comprises a hydrophobic fusion loop (E1-FL) that inserts into the host membrane during the fusion process. Under low-pH conditions, E1 experiences conformational alterations, which result in the exposure of the fusion loop and facilitate the merger of membranes. Crucial residues such as E1-A/V226 and E1-V178 are instrumental in lipid sensing and fusion efficiency [170,181]. For instance, the E1-V178 residue is significantly conserved among most alphaviruses. Experimental mutation of this residue to alanine has been shown to result in a reduced dependence on cholesterol for CHIKV fusion [194].

E2 glycoprotein encompasses three structural domains, designated as A, B, and C. Domain A, centrally located, is surface-exposed and facilitates interactions with host receptors, such as matrix remodeling-associated protein 8 (Mxra8). Domain B, situated distally, encompasses the acid-sensitive region (ASR) and serves as a primary target for neutralizing antibodies. Domain C, which is embedded within the viral membrane, anchors E2 and exhibits reduced accessibility to immune detection [167,192].

The CP interacts with the cytoplasmic tail of E2, thereby facilitating the assembly and envelopment of the nucleocapsid. During the viral entry process, the capsid remains associated with the viral RNA, constituting the nucleocapsid core. After membrane fusion, the nucleocapsid is released into the cytoplasm, where the uncoating process occurs. The capsid disassembles, releasing the viral RNA, which is then available for replication [151,192].

#### 3.1.2. Host Cell Receptors and Attachment Factors

The primary receptor for CHIKV is Mxra8, which interacts with domains A and B of the E2 protein. This interaction promotes the entry of the virus into fibroblasts, myocytes, and osteoblasts. Mxra8 functions as a conserved receptor across numerous alphaviruses, thereby establishing its relevance as a prospective target for therapeutic intervention [167,195]. Another receptor, Prohibitin-1 (PHB1), has been shown to interact specifically with E2 in certain cell types, including microglial cells. While PHB1 plays a role in facilitating viral attachment, it is not essential for viral entry, suggesting its function may be to enhance the concentration of virions at the cellular surface [193].

Glycosaminoglycans (GAGs) represent substantial, intricate carbohydrate macromolecules on the extracellular surfaces of diverse mammalian cell types. Notably, heparan sulfate plays a pivotal role as an attachment factor, facilitating the enhancement of viral attachment to the cellular surface [196]. These macromolecules can interact with various proteins and principally serve functions in cellular adhesion, proliferation, differentiation, and signal transduction [192]. Mutations in E2, including E2-R82, enhance GAG affinity, facilitating adaptation to cell culture while decreasing virulence in vivo [192,197,198].

Phosphatidylserine (PtdSer) receptors, such as T-cell immunoglobulin and mucin domain (TIM-1), the TAM family proteins (comprising Tyro3, Axl, and Mer), and CD300a, are capable of recognizing PtdSer present on the viral envelope [199,200]. These receptors enable entry by mimicking apoptosis, but they do not trigger the conformational changes necessary for fusion [181,201].

#### 3.1.3. Mechanisms of Membrane Fusion

CHIKV enters host cells via clathrin-mediated endocytosis, facilitating the delivery of virions to early endosomes. The subsequent acidification of these endosomes, reaching a pH of approximately 6.0, triggers conformational alterations in the E1 protein, leading to the exposure of the hydrophobic fusion loop (E1-FL) [202]. This fusion loop is then inserted into the host cellular membrane, thereby initiating the process of membrane merger. Additionally, the presence of cholesterol and sphingomyelin within the host membrane significantly enhances the efficiency of this fusion process [151,193,203].

At low pH levels, E2 dissociates from E1, thereby facilitating the trimerization of E1 and the insertion of the fusion loop [202]. The ASR within E2, particularly the E2-H170 residue, contributes to the destabilization of the heterodimer. Furthermore, E3, which provides stability to E2 during the viral assembly process, is cleaved during viral maturation, consequently rendering the virus competent for fusion [167,192,204].

CHIKV demonstrates the ability to fuse with receptor-free liposomes, indicating that this fusion process is independent of any protein receptor. This fusion mechanism can be delineated into several distinct stages: the destabilization of the E2/E1 heterodimer, integrating the E1 protein into the target membrane, the trimerization of E1, and the subsequent formation of the fusion pore [205].

In summary, E1 trimers assemble into a ring-like structure on the host membrane, facilitating the proximity between viral and host membranes. This interaction leads to hemifusion, succeeded by the establishment and subsequent enlargement of a fusion pore. Consequently, the nucleocapsid is liberated into the cytoplasm, initiating the processes of uncoating and replication [170,206,207,208].

#### 3.1.4. Endocytic Pathways Utilized by Chikungunya Virus

CHIKV primarily enters host cells through clathrin-mediated endocytosis (CME), a well-characterized and constitutive process in mammalian cells [209,210]. CME involves the formation of clathrin-coated pits at the plasma membrane, which encapsulate the virus. This process is mediated by a complex interplay of proteins, including adaptor protein-2 (AP-2), clathrin, dynamin, and epidermal growth factor receptor substrate 15 (Eps15) [209,210]. Dynamin, a large multidomain guanosine triphosphatase (GTPase), plays a critical role in the scission of clathrin-coated pits, leading to the formation of clathrin-coated vesicles that transport the virus into the cell [209,210]. Once internalized, the clathrin coat is rapidly removed, and the virus is delivered to early endosomes [193,211]. The acidic environment of the endosomes triggers conformational changes in the viral E1/E2 glycoproteins, facilitating the fusion of the viral envelope with the endosomal membrane and the subsequent release of the viral genome into the cytoplasm for replication [181].

Dynamin is a key mediator of CME and is essential for the pinching of endocytic vesicles from the plasma membrane. Its role in CHIKV entry has been well-documented, as inhibition of dynamin significantly reduces CHIKV infection [11,212,213]. Similarly, Eps15, a protein critical for the assembly of clathrin-coated pits, has been implicated in CHIKV entry. However, the involvement of Eps15 does not exclusively confirm CME, and studies have shown that CHIKV can enter cells via a clathrin-independent, Eps15-dependent pathway. They demonstrated this using knockdown of Eps15 and clathrin heavy chain, a major scaffold protein of the clathrin coat [214].

These findings indicated that the entry of the CHIKV is not exclusively limited to CME; rather, it may also engage other endocytic mechanisms that depend on the cellular context. CHIKV’s ability to utilize multiple entry pathways highlights the flexibility of its infection strategy and underscores the necessity for further research to thoroughly elucidate these underlying processes.

## 4. Immunological Aspects of Chikungunya Virus Infection

In this section, we will explore the inflammatory processes triggered by the host immune response following CHIKV entry. Focus will be given to the roles of cytokines, chemokines, and immune cells in modulating viral load—either promoting viral clearance or persistence—as well as the clinical symptoms resulting from these immunological mechanisms. The key aspects related to these responses are outlined below.

The acute phase is marked by high viral replication and dissemination from the primary sites of CHIKV infection, resulting in a peak in viremia and the manifestation of clinical symptoms characteristic of the acute phase [115]. The main target organs during infection include the liver, spleen, joints, and kidneys, allowing the infection of non-hematopoietic cells such as fibroblasts and endothelial cells. CHIKV replication is recognized by pattern recognition receptors (PRRs), such as NOD-like receptors (NLRs) and Toll-like receptors (TLRs), resulting in the downstream activation of nuclear factor kappa B (NF-kB) and the phosphorylation of interferon regulatory factor (IRF) 3, initiating the production and release of Type I Interferon (IFN-I) as well as other cytokines. Thus, at the site of skin infection, resident immune cells, such as lymphocytes, DCs, monocytes, and gamma delta T lymphocytes (γδ T cells), recognize the viral particles through PRRs and induce a rapid and robust production of IFN-I (Interferon alpha and beta [IFN-α/β]), along with other cytokines that will promote the additional recruitment of immune cells and the activation of adaptive immunity, essential for viral control and elimination. Consequently, with the release of chemoattractant molecules by resident cells, such as C-C motif chemokine ligand (CCL2)/MCP-1, there is an expressive migration of macrophages, neutrophils, natural killer (NK) cells and lymphocytes to the primary sites of infection (e.g., joints and muscles), leading to hypertrophy of synovial cells and adjacent synovial vessels [215], and subsequent joint pain in patients [115,216]. There is also the development of adaptive immunity, with a CD8+ T lymphocyte response in the early stages of infection and, later, the development of a CD4^+^ T cell response [217]. In addition, from the 3rd day after the onset of clinical symptoms, immunoglobulin (Ig) M antibodies for CHIKV can also be detected [218,219,220]. The chronic phase lasts longer than 3 weeks and can extend from months to years. It is characterized by signs and symptoms such as joint swelling, stiffness, arthralgia, and tendonitis/tenosynovitis. In this stage, there is a significant reduction in viral titers. However, viral particles can remain in macrophages and fibroblasts, since CHIKV can use these cells as replication reservoirs [121]. Pain is one of the most critical symptoms of the chronic phase of CHIKF. Some studies have shown that the E2 envelope protein induces the humoral immune response [221] and maturation of CD4^+^ T lymphocytes in patients [222], resulting in inflammation and swelling of the joints. In addition, the E2 protein is also involved in CHIKV-induced joint pain. Recently, Segato-Vendrameto et al. (2023) [223] demonstrated that the E2 protein activates dorsal root ganglion (DRG) neurons, resulting in calcium influx via Transient Receptor Potential Vanilloid 1 (TRPV1) and neuronal sensitization. As a result, the E2 protein leads to the development of mechanical and thermal hyperalgesia, which was reversed by the use of monoclonal antibodies directed against the E2 protein.

### 4.1. Innate Response

In CHIKV infection, the innate immune response contributes to antigen recognition, limitation of viral replication, and creating a microenvironment towards mounting an effective adaptive response. These functions are performed by immune cells such as dendritic cells, neutrophils, monocytes, macrophages, and NK cells. When activated, these cells begin to produce IFN-α, IFN-β, Interferon gamma (IFN-γ), tumor necrosis factor (TNF)-α, IL-6, IL-β, for example, which act to orchestrate the mechanisms of viral elimination. Next, we will explore important aspects of these cells in CHIKV infection.

DCs are resident antigen-presenting cells that capture, process, and present CHIKV antigens to T lymphocytes, initiating specific antiviral immune responses. Dermal DCs are the main activators of the IFN-I response to CHIKV and are primarily responsible for limiting viral replication and clinical progression [224]. The IFN-I signaling pathway is extremely important for controlling CHIKV infection, as evident using knockout (KO) animals and by genetic deletion of myeloid differentiation primary response gene 88 (MyD88) that resulted in increased viremia in mice [225,226,227]. In addition, when DCs are exposed to CHIKV, the activation of PRRs leads to the activation of signaling pathways, such as IFN-I and NF-kB, resulting in the activation of IκB-α and the release of TNF-α and IL-12p70 in CD11c^+^ CD86^+^ DCs, and increased production of IL-2 in CD4^+^ T cells [228]. The activation of these pathways and subsequent cytokine production contribute significantly to viral control and elimination from the host. In addition, Long et al. (2013) [229] demonstrated the functional importance of the dendritic cell immunoreceptor (DCIR) in arthritis triggered by CHIKV, suggesting CHIKV infection. In the study, the inhibition or absence of the receptor in DCs exposed to virus increased the expression of IL-10 and IL-6, and reduced IL-12, resulting in more severe disease (marked leukocyte infiltration, joint swelling and damage, and loss of body weight). In addition, receptor deficiency leads to delayed clearance of CHIKV [229].

Neutrophils are cells recruited by the onset of CHIKV infection. Neutrophil migration occurs through the production of chemokine (CXC motif) ligand (CXCL) 1 and CXCL2, and once at the site of infection, these cells can produce reactive oxygen species (ROS), cytokines (e.g., IFN-I), and neutrophil extracellular traps (NET), which contribute to the process of controlling acute CHIKV infection [230,231,232]. In these cells, IFN-I (IFN-α and IFN-β) signal through the same receptor, the IFN α/β receptor (IFNAR), but they have distinct functional mechanisms in the antiviral response against CHIKV. The KO mice demonstrated that IFN-α is essential for controlling viral replication and spread, and IFN-β acts primarily in modulating the inflammatory response [233]. Furthermore, the increase in neutrophils led to greater tissue damage at infected sites resulting in increased production of pro-inflammatory cytokines and chemokines (e.g., TNF-α, IL-6, CCL2, and CXCL1), edema, pain, and tissue damage, indicating that these cells not only aided in defense but also contributes to the worsening of immunopathology when present in excess [234]. Moreover, NETs induced in neutrophils in vitro were able to capture and neutralize CHIKV. In vivo (animal model), NET release depends on the activation of TLR7 and ROS generation. In patients with acute CHIKV infection, increased levels of the myeloperoxidase (MPO)-DNA complex (a NET marker) were detected, and there was a correlation between these levels and viral load in the blood. All these findings reinforce that NETs play an essential protective role in controlling the acute phase of CHIKV infection [232,235]. On the other hand, in musculoskeletal tissues during CHIKV infection, anti-inflammatory neutrophils (N2-subtype) infiltrates to modulate the inflammation. Consequently, it may compromise viral clearance and delay disease resolution [236]. Thus, these sets of cells are important for the initial control of the infection, but in the long term, neutrophil activity can contribute to the degree of the chronic phase of the disease [237,238].

Monocytes play an important role in restricting CHIKV infection, as their presence in synovial tissue is associated with elevated expression of IFN-α, which can inhibit viral replication [116,239,240]; and play an important role in the development of joint pathology [241]. Monocyte-derived macrophages migrate to the site of infection in response to the activation on chemoattractants, such as CCL2/MCP-1, induced by CHIKV [129,242]. Both resident and infiltrating macrophages produce IL-6, TNF-α, and granulocyte macrophage colony stimulating factor (GM-CSF), contributing to local inflammation [115,243]. Furthermore, these cells are susceptible to CHIKV infection and can act as viral reservoir and sites of replication with infiltration of NK cells and CD4^+^ T lymphocytes, which also contributes to the perpetuation of pain and polyarthralgia [221]; and induce antiviral control and the development of chronic arthritis due to IFN-α production [116,119,129,221,244]. The role of monocytes and macrophages is therefore dual. The inhibition by a monocyte chemotactic protein inhibitor (e.g., CCL2/MCP-1 and CCL8), reduce in osteoclastogenesis, preventing bone resorption [245]; and their depletion can result in increased neutrophil infiltration into joints, resulting in tissue damage and pain [245,246,247]. Furthermore, CHIKV can directly infect human osteoblasts, which increased expression of IL-6, activation of receptor activator of nuclear factor kappa-B ligand (RANKL), and inhibition of osteoprotegerin (OPG), which contributes to bone loss [248,249]. This dysregulation, associated with impaired osteoblast function, contributes to increased alkaline phosphatase levels, directly affecting bone mineralization [250]. Macrophages are essential sources of CCL2, regulating the migration of monocytes and NK cells to the site of infection [242,247]. However, a decreased monocytes/macrophages infiltration results in a compensatory neutrophil and eosinophil response, which can contribute to edema and tissue damage [247]. Therefore, targeting these cells requires a balanced approach to control both viral persistence and inflammation.

In CHIKV infection, the peak of NK cells occurs during the initial acute phase, around 3 days after the onset of symptoms, and correlates directly with viral load; and the persistence of this cell is associated with the progression into the chronic phase [251]. NK cell function is regulated by several classes of receptors expressed on their cells membranes, such as lectin C-type receptors [e.g., NKG2D (activating) and CD94/NKG2A (inhibiting)], killer cell immunoglobulin-like receptors (KIRs) [KIR2DS, KIR3DS, and their inhibitors KIR2DL and KIR3DL—activating and inhibitory variants that recognize human leukocyte antigen (HLA) and regulate the immune response], and natural cytotoxicity receptors (NCRs) (e.g., NKp30, NKp44, and NKp46, which detect cellular stress markers or viral antigens) [252]. The coordinated expression of activating and inhibitory receptors allows NK cells to respond in a balanced manner [252]. It has been observed that an imbalance of these receptors is associated with increased susceptibility of patients to CHIKV infection [253,254,255]. A high viral load in the acute and chronic phase can be associated with different NK cells (e.g., CD69^+^), and the release of cytokines like TNF-α and IFN-γ, and the persistence of these factors being correlated with persistent arthralgia [254,256,257]. The elevated expression of CD3^−^ CD56^+^ NK cells, and expression of active NKG2C receptor and KIR2DL2/KIR2DL3 inhibitory receptors for HLA-C1 are related to higher viremia and the clearance of infected cells in the acute phase [241]. Also, the increase in the frequency of HLA-C2, which presents peptides to CD8^+^ T cells in combination with the expression of KIR2DL1 gene (receptor for HLA-C2), can worsen the infection [253,258]. Other studies have demonstrated that, after acute CHIKV infection, NK cells undergo a transient clonal expansion linked to increased viral load [251,254]. On the other hand, due to their cytotoxic activity, NK cell expansion may contribute to joint pathology [256]. Overall, NK cells contribute both to viral clearance and, potentially, to disease chronicity.

The innate immune response to CHIKV infection is detailed below and summarized in Table 1.

### 4.2. Adaptative Immune Response

The adaptive immune response occurs via the activation of CD4^+^ and CD8^+^ T lymphocytes, which promote the elimination of infected cells and the secretion of cytokines (IFN-γ, IL-2, and TNF-α) that will amplify the antiviral functions of immune cells such as macrophages. In addition, the activation of B lymphocytes results in the production of neutralizing antibodies (Immunoglobulin (Ig)M and IgG) against CHIKV.

The role of T cells in the pathogenesis of alphaviruses infections is varied [274,275,276,277]. In fact, during the early phase of CHIKV infection, there is a predominance of CD8^+^ T cells, which are responsible for antiviral immunity, cytotoxic activities, and the destruction of infected cells to control virus replication, and those cells remain in the blood for 7–10 weeks post-infection [217,264,265]. Towards the end of the acute phase, the CD4^+^ T cell is responsible for modulating the activity of other immune cells—by producing IFN-γ, they stimulate cell-mediated and production of neutralizing antibodies [263]. Furthermore, an increase in the frequency of CD4^+^ T cells observed in patients with persistent CHIKV-associated arthralgia suggests a role in antiviral defense [278] and in inducing joint damage [221,222,262,279] by mechanism mediated by regulatory T cells (Tregs) and T cells, such as types I (Th1) and type 17 (Th17) helper T (Th) cell subsets [217,222,256,262,263,280].

During CHIKV infection, Th1 has promoted cell-mediated immunity, being able to activate macrophages to fight intracellular pathogens, producing cytokines like IFN-γ and TNF, and enhancing the cytotoxic activity of both NK cells and CD8^+^ T cells [266]. On the other hand, Th2 is involved mostly in humoral response, promoting B cell stimulation through IL-4, IL-5 and IL-13 signaling, and modulating Th1/Th17 inflammatory responses [256]. Th17 cell subsets can act in concert with Th1 or alone, increasing the production of the cytokines IL-17, IL-6, IL-21, and IL-22, and playing important roles in inflammation and tissue damage related with joint and muscle pain [263,270,281,282,283,284,285,286]. Treg cell activity decreases in parallel with the reduction in viral load, as their specific function protects against CHIKV-induced pathology in mice [263].

Cells infected with CHIKV are killed mainly by the action of cytotoxic immune cells, such as CD8^+^ T lymphocytes. Once activated, effector CD8^+^ T lymphocytes promote release of cytolytic granules through exocytosis or the granule-independent pathway, resulting in cytotoxicity, and produce important antiviral cytokines (e.g., IFN-γ) [284].

T cells play a complex role in CHIKV infection. CD8^+^ T cells dominate early, contributing to viral clearance through cytotoxicity and IFN-γ production, but may become exhausted during chronic infection, reducing their effectiveness. CD4^+^ T cells support immune coordination and antibody production but can also drive joint inflammation, especially through Th1 and Th17 responses. The expansion of these subsets, along with decreased Treg cell activity, is linked to tissue damage and chronic symptoms. Overall, while T cells are crucial for antiviral defense, their dysregulation may contribute to chronic CHIKV-associated pathology.

B lymphocytes and antibodies specific to CHIKV are essential for limiting viral replication and dissemination [129,273]. In addition, a study using serum samples from CHIKV-infected humans in Malaysia demonstrated that B cells produce neutralizing IgM antibodies targeting CHIKV surface glycoproteins E1 and E2 at day 6 post-infection, and the production of these antibodies is associated with lower levels of viremia [272]. Another study using human samples showed that CHIKV infection results in the production of IgG and the generation of memory B cells that can last up to 24 years after infection [271]. Furthermore, Hoarau et al. (2010) [221] demonstrated that E1/E2 and capsid proteins are the main factors that drive the humoral immune response in hospitalized patients with CHIKV, leading to increased production of IFN-γ and IL-12 and activation of cells such as NK cells and memory/effector B lymphocytes, these appear to have driven tissue damage, apoptosis, fibrosis and a polarized inflammatory response in CHIKV-induced arthritis, especially due to the persistence of viral RNA in synovial macrophages. CHIKV can elicit cellular and humoral immune responses through envelope and capsid proteins, contributing, for example, to the development of arthritis [221].

B cells and CHIKV-specific antibodies are critical for controlling viral replication and spread. In B-cell-deficient mouse models, infection leads to higher and persistent viremia, highlighting the importance of humoral immunity. Neutralizing IgM antibodies targeting CHIKV E1 and E2 glycoproteins appear early and are associated with reduced viremia, while long-term IgG and memory B cell responses can persist for decades. Despite this, chronic arthritis may occur independently of adaptive immunity, driven instead by viral persistence in tissues. Additionally, aging impairs B cell responses, contributing to prolonged infection and higher viral loads in elderly hosts.

The adaptive immune response to CHIKV infection is detailed below and summarized in Table 2.

## 5. Diagnosis and Clinical Management for Chikungunya Virus

**Figure 5:**
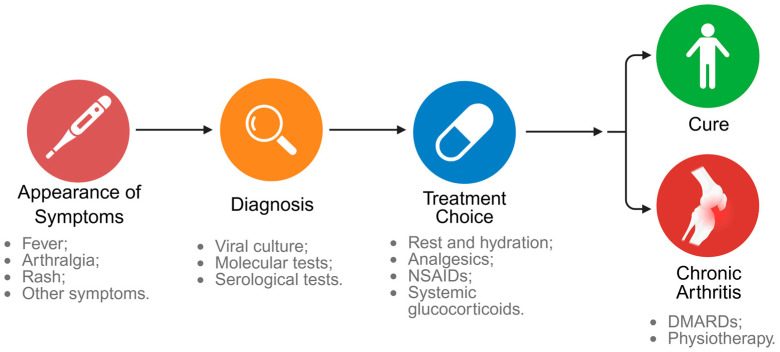
Clinical evolution of Chikungunya infection. After the appearance of clinical signs such as fever, arthralgia and rash, the diagnosis is confirmed through viral culture, molecular or serological tests. Treatment is based on symptomatic relief with rest, hydration, analgesics, NSAIDs, and, in selected cases, systemic glucocorticoids. A clinical course can lead to cure or progression to chronic arthritis, which requires interventions such as the use of DMARDs and physical therapy. Created in BioRender. Oliveira, F. (2025) https://BioRender.com/15wrcvb (accessed on 14 July 2025).

The diagnosis of CHIKV infection is based on a combination of clinical, epidemiological, and laboratory data, and is essential to guide the initiation of treatment, which is currently limited to symptomatic relief and supportive therapies. Figure 5 summarizes the main diagnostic methods and treatments indicated for CHIKV.

Initially, clinical evaluation is based on the presence of classic symptoms such as sudden-onset fever (39 to 40 °C), severe and often debilitating arthralgia (reported in 85–90% of cases), rash (reported in 40–60% of cases), and other flu-like symptoms (e.g., headaches and gastrointestinal discomforts) [299]. Although the symptoms are nonspecific and similar to those of infections by other arboviruses, such as dengue virus (DENV) and zika virus (ZIKV), these signs may suggest a CHIKV infection [300]. The conjunctive presence of these characteristic symptoms is highly predictive of CHIKV infections in endemic regions [300]. However, specific laboratory diagnosis is necessary to confirm CHIKV infection, especially in regions where other arboviruses are endemic [5].

CHIKV infection can be diagnosed through detection of the virus itself, the viral genome or virus-specific antibodies, using viral culture, molecular, and serological tests [299,301]. Although viral isolation was considered the gold standard for viral detection for decades, these methods are now more commonly used in research rather than clinical diagnosis. This is due to the long time required to perform the test and the need for specialized equipment and trained professionals [299]. Among the molecular diagnostic methods, reverse transcription polymerase chain reaction (RT-PCR) is the most sensitive and is currently considered the gold standard for the detection of viral RNA [11,299,301]. However, RT-PCR is only effective during the acute phase of infection (within 5 to 7 days of symptom onset), where the viral load can reach levels high enough for detection [11,301]. Other molecular tests include isothermal methods and multiplex assays [299].

After the acute phase of CHIKV infection, serological tests that identify IgM and IgG antibodies are most commonly used, since IgM are detectable in the early stages of the disease and IgG are detectable for a long-time post-infection [299,301]. Among the serological tests, enzyme-linked immunosorbent assays (ELISA) are the most widely used due to their ability to detect antibodies produced during CHIKV disease several months after the initial infection [11,299]. However, although IgM and IgG antibodies to CHIKV are highly sensitive, they can cross-react with other alphaviruses [299,302], such as DENV, Mayaro, and o’nyong-nyong, leading to possible misdiagnosis [303,304] and requiring a more specific neutralization test to confirm the results.

Currently, there are no specific treatments for CHIKV infection; therefore, therapeutic strategies are based on supportive treatment, including pain management and anti-inflammatory medication. During the acute phase of infection, the recommended treatment is rest, adequate hydration, and analgesics, with acetaminophen being the first-line medication. Nonsteroidal anti-inflammatory drugs (NSAIDs), such as ibuprofen, can also be used, except in cases where dengue co-infection is suspected. The indicated analgesics and NSAIDs, as well as supportive therapies (e.g., hydration and rest), relieve fever and muscle and joint pain. However, when pain is not controlled with commonly used analgesics, more potent analgesics may be indicated [11,305]. Studies showed that broad-spectrum antivirals, such as ribavirin (RBV) and IFN, are effective against CHIKV, however more research on these drugs is needed for clinical implementation [5].

In cases of arthralgia or severe joint inflammatory responses, systemic glucocorticoids may be indicated. However, when symptoms progress to chronic arthritis, inflammatory arthritis treatment protocols are recommended, including disease-modifying antirheumatic drugs (DMARDs), such as methotrexate (MTX), which are indicated to slow the progression of the disease. Together, physiotherapy is an important ally in the rehabilitation of patients with chronic post-CHIKV manifestations [305]. A retrospective study conducted in a Spanish center analyzed 119 patients with post-CHIKV complications, where the main clinical manifestation was persistent arthralgia (86% of patients). Management with targeted therapies resulted in clinical improvement in chronic cases, highlighting the importance of early diagnosis, specialized follow-up, and the adoption of interdisciplinary protocols to optimize treatment [306].

## 6. Emerging Therapies for Chikungunya Virus Infection

**Figure 6:**
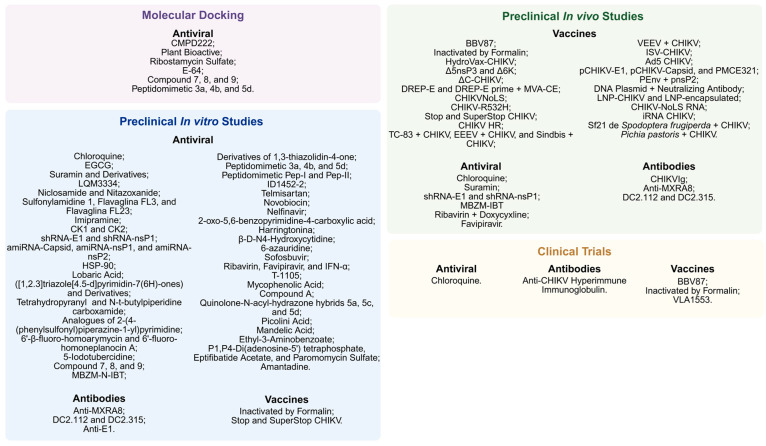
Main therapeutic strategies under investigation against Chikungunya virus. Treatments are distributed according to their stage of development: Molecular Docking, in vitro and in vivo preclinical studies, and clinical trials. Approaches include antiviral compounds, antibodies, and experimental vaccines, highlighting the diversity of targets and mechanisms evaluated in combating CHIKV infection. Some treatments are identified by their acronym, and their full names can be found in topic 6. Created in BioRender. Oliveira, F. (2025) https://BioRender.com/15wrcvb (accessed on 18 August 2025).

The growing disease burden and the risk of global outbreaks, coupled with the lack of specific treatments, have driven the development of targeted therapies. Emerging therapeutic strategies seek to interfere with viral replication, modulate the host immune response, or attenuate long-term inflammatory complications [307]. These approaches include antiviral agents, polyclonal and monoclonal antibodies, and therapeutic vaccines, many of which are still in preclinical phases or in early clinical trials [308]. This section and Figure 6 summarize the main therapeutic approaches under development against CHIKV.

### 6.1. Antiviral Agents

Over the past decade, the development of specific antiviral agents against CHIKV has been the subject of intense research. However, it is important to emphasize that to date there are no licensed antiviral agents that act directly against CHIKV, and most studies are in preclinical phases [307]. Table 3 summarizes the main antiviral agents in preclinical and clinical development against CHIKV. Below, we detail only the viral agents in preclinical development in vivo (animal models) and at the clinical stage.

#### 6.1.1. Entry and Fusion Inhibitors

The entry and fusion of CHIKV into host cells is a key event for the establishment of infection. Thus, inhibiting this initial step represents a promising strategy in the development of antivirals, since it prevents the multiplication of the virus and contributes to the reduction in viral load, inflammatory response, and clinical symptoms of the disease [364].

Among the antiviral agents, Chloroquine, a drug indicated for the treatment of malaria, systemic lupus erythematosus and rheumatoid arthritis [365], was the target of major investigations. However, in clinical phase studies, the treatment has not shown any benefit to the patient. Roques et al. (2018) demonstrated that in animal models, prophylactic administration of Chloroquine aggravated the infection by increasing viremia and slowing the immune response, while in humans, treatment modulated the levels of inflammatory markers with the potential to allow the adaptive immune response [310]. Other studies with similar effects are described in Table 3.

Suramin, a Food and Drug Administration (FDA)-approved compound for the treatment of trypanosomiasis, acts as a competitive inhibitor of GAGs and has been investigated for the treatment of CHIKV. Studies have shown that Suramin works by dose-dependent reduction in cytopathic effect, viremia, protein expression, and viral production when administered early [314]. Its mechanism of action involves direct interaction with envelope glycoproteins, blocking conformational changes necessary for viral binding and fusion [366]. In vivo studies, Suramin decreased viral loads, improved acute lesions, restored cartilage integrity, and reduced the number of chondrocytes in infected mice, confirming its role as a multifunctional inhibitor in early stages of CHIKV infection [316]. Other studies are presented in Table 3. However, despite promising results from different studies, clinical trials involving patients with hepatitis B [367] and acquired immunodeficiency syndrome (AIDS) [368] suggest that long-term treatment with this antiviral is directly related to the appearance of serious side effects.

#### 6.1.2. RNA Interference

Interference RNAs (RNAi) are biological molecules that regulate gene expression. SiRNA promotes gene silencing by cleaving the corresponding messenger RNA (mRNA), preventing protein production. This mechanism has been explored as a potential antiviral therapy, being able to act both directly against the virus and in host cell genes [369]. Lam et al. (2012) developed three small loop interfering RNA (shRNA) sequences targeting the *capsid*, *E1* and *nsP1* genes of CHIKV [324]. The authors observed that shRNA-containing cells against E1 and nsP1 showed total suppression of virus production until the third day post-infection, while the shRNA directed to the capsid showed moderate inhibitory effects. The shRNA targeting the E1 protein blocked several geographic strains of CHIKV, without affecting the replication of other viruses (e.g., DENV and SINV) indicating high specificity. In addition, it has been shown to be effective in an in vivo model, fully protecting mice from CHIKV infection [324].

#### 6.1.3. Non-Structural Protein Inhibitors

The nsP2 protein of CHIKV exerts multiple essential functions in viral replication, acting as protease, helicase, nucleoside-triphosphatase (NTPase), RNA triphosphatase (RNAse), and virulence factor by suppressing the host immune response via transcriptional shutdown [369]. In addition to its essential role in CHIKV replication, the early discovery of the 3D structure of nsP2 has made it the target of investigations for the development of anti-CHIKV drugs [364].

Mishra et al. (2016) investigated the antiviral activity of the compound 1-[(2-methylbenzimidazole-1-yl)methyl]-2-oxo-indolin-3-ylidene]amino]thiourea (MBZM-N-IBT) against CHIKV [340]. MBZM-N-IBT reduced plaque-forming unit (PFU) formation approximately 76% and viral RNA levels of nsP2 and E1 by approximately 65% and 24%, respectively. In addition, the viral protein expression of E2 and nsP2 were inhibited by 97%, indicating strong blockade of viral replication and translation. In summary, MBZM-N-IBT showed potent antiviral activity against CHIKV, with low toxicity and inhibition in the early and late phases of infection [340].

NsP4 is an essential polymerase for alphaviruses, responsible for the synthesis of viral RNA. Its C-terminal domain contains the typical structure of an RNA-dependent RNA polymerase (RdRp), with GDD motif at the active site. Because nsP4 is absent in humans and has conserved regions between several viruses (such as CHIKV, ZIKV, and DENV), it is a promising target for the development of broad-spectrum antivirals [369].

Nucleoside analogues (NAs) are synthetic molecules that mimic natural nucleosides and, upon intracellular activation by phosphorylation, can inhibit the synthesis of viral DNA or RNA, disrupting replication [370]. Ferreira et al. (2019) demonstrated that oral treatment with Sufosbuvir administered just before CHIKV infection significantly reduced paw edema in adult mice [352]. The treatment of newborn mice increased the survival of infected animals and prevented CHIKV-induced motor neuron impairment. After investigating the interaction between Sufosbuvir and the nsP4 enzyme, the authors identified that there were plausible hydrogen bonds and electrostatic interactions that underpin the potential mechanism presented by the treatment [352].

In addition, Franco et al. (2018) compared the activity of three broad-spectrum antiviral agents, including two NAs and IFN-α (Table 3), against CHIKV in different cell lines [353]. RBV, a guanosine analogue, inhibited viral RNA synthesis and depleted intracellular GTP by interference with inosine monophosphate dehydrogenase (IMPDH), but was of little promise when administered alone due to high toxicity at effective concentrations [353]. Other studies have investigated the mechanisms of action of RBV and proposed that it acts by directly inhibiting the nsP4 RdRp protein through interaction with the Cys483 residue [371] and that the combined administration of RBV with Doxycycline [354] or IFN-α [355] is effective against CHIKV infection without showing toxicity.

Franco et al. (2018) also demonstrated that Favipiravir (FAV or T-705), a purine analogue, acted as a substrate for CHIKV’s RdRp, promoting lethal mutagenesis and chain termination, resulting in inhibition of viral replication [353]. In addition, the compound showed good antiviral selectivity and low toxicity in human cells, suggesting its potential for clinical studies [353]. Together, Delang et al. (2014), investigated the action of FAV and identified that the K291R mutation in the highly conserved F1 motif of the RdRp of the +ssRNA viruses (*nsP4 gene*) is responsible for the resistance of CHIKV to the drug, pointing to this enzyme as a relevant molecular target [330]. Similar results were observed with the defluorinated analog of the FAV (T-1105) [330]. However, the effectiveness of treatment depends on the timing of administration: FAV is highly effective when used in the early stages of infection, preventing the spread of the virus to distant joints and tissues. Administration after this period has low efficacy against CHIKV infection [356].

#### 6.1.4. Host-Directed Antivirals

Host factors can favor or inhibit viral replication and, therefore, are promising targets for the development of antivirals. However, as they also participate in essential functions of the body, their modulation can cause toxicity. Thus, ideally, therapies should specifically target virus–host interactions, without affecting vital cellular processes. A thorough and detailed review on host-directed antivirals is presented by Hucke and Bugert (2020) [369], Battisti et al. (2021) [364] and Haese et al. (2022) [372].

### 6.2. Antibodies

The use of antibodies represents a promising therapeutic strategy for the control of emerging and reemerging viral infections. Polyclonal antibodies (pAb) are responsible for recognizing multiple epitopes of the same antigen and are historically used in immune replacement therapies and in passive immunizations (e.g., against hepatitis A, rabies, and measles viruses). Monoclonal antibodies (mAbs), on the other hand, are responsible for recognizing a single epitope of the antigen and offer advantages in terms of safety, specificity, and pharmacological potential [373]. Table 4 summarizes the main studies in preclinical and clinical development with polyclonal and monoclonal antibodies against CHIKV. Below, we discuss in detail only the antibody therapies in preclinical development in vivo (animal models) and in the clinical phase.

#### 6.2.1. Polyclonal Antibodies

Couderc et al. (2009) investigated the use of human polyclonal immunoglobulins (CHIKVIg), extracted from individuals in the convalescent phase of CHIKV infection, in murine models [374]. Plaque reduction neutralization tests (PRNT) showed that the serum containing CHIKVIg has a high viral neutralization capacity. In adult mice deficient (^−/−^) for IFN-α/βr, administration before or shortly after CHIKV infection prevented the disease, which was confirmed by the absence of viremia and clinical signs. In addition, administration to CHIKV-infected newborn mice significantly reduced mortality, viral load, and symptoms. These results demonstrated that purification of antibodies against CHIKV from convalescent plasma is effective and can be considered as a strategy to prevent and treat CHIKV infections [374].

In addition to preclinical trials, phase I and II clinical trials have been conducted to evaluate the safety and tolerability of administering anti-CHIKV hyperimmune intravenous immunoglobulin (derived from plasma from convalescent individuals) to newborns via vertical transmission, as well as to select the safe and effective dose for the development of a new intervention. To date, there are no scientific publications that present the clinical results of this study [ClinicalTrials.gov: NCT02230263].

#### 6.2.2. Monoclonal Antibodies

Through a Clustered Regularly Interspaced Short Palindromic Repeats (CRISPR)-Cas9-based genomic screening, the cell adhesion molecule Mxra8 was identified as a mediator of CHIKV and other alphaviruses, binding to the A and B domains of the E2 protein. CHIKV virions bind directly to Mxra8, allowing viral attachment and internalization by the cell. In vivo, blocking Mxra8 reduced viral load and clinical signs of infection [195]. In addition, it was identified that mAbs isolated from individuals exposed to CHIKV were protective because they prevented virions from binding to the Mxra8 receptor. Structural assays have revealed that antibodies bind to a conserved epitope on viral proteins, directly obstructing the interaction interface with Mxra8. These findings pave the way for the development of new antiviral strategies against CHIKV [377].

Two other mAbs isolated from individual’s plasma in the convalescent phase of CHIKV infection were characterized, namely DC2.112 and DC2.315. These antibodies bind to a highly conserved epitope of the E1 protein, allowing cross-neutralization of several alphaviruses. As a result, these antibodies showed weak neutralization, but significantly inhibited the viral release of infected cells, suggesting a new mechanism of viral control. They were also able to recruit myeloid cells to promote phagocytosis of infected cells. In vivo, both mAbs conferred robust protection against CHIKV infection, highlighting the E1 protein as a promising new target for the development of therapeutics and vaccines [375].

### 6.3. Vaccines

Several scientific efforts have been directed to the development of effective vaccines against CHIKV, aiming to prevent the infection and contain its spread in endemic areas. There are currently several vaccines against CHIKV in the preclinical and clinical phases, which include inactivated, chimeric, recombinant, DNA or RNA-based, VLP and live attenuated vaccines (LAV) vaccines [4]. Table 5 summarizes the main vaccines in preclinical and clinical development against CHIKV. Below, we focus on detailing only those vaccines currently in clinical development.

The BBV87 vaccine was developed from the CHIK/03/06 strain (ECSA genotype, originating in India, 2006) cultured in Vero cells and inactivated by β-propiolactone (BPL). Phase I and II clinical trials demonstrated that healthy adults who received 2 or 3 doses of the vaccine had 100% seroconversion and a robust neutralizing antibody response that was maintained for at least 6 months. In addition, the vaccine was well tolerated, and adverse events were mild and self-limited [378]. Phase II and III clinical trials were conducted together to evaluate the safety and immunogenicity of two doses of the BBV87 vaccine in three endemic countries in Asia and Latin America, as well as to select the optimal dose for the confirmatory phase of the study. To date, there are no scientific publications that present the clinical results of this study [ClinicalTrials.gov: NCT 04566484].

Harrison et al. (1971) described the production and evaluation of a formalin-inactivated vaccine for CHIKV [379]. To produce the vaccine, CHIKV was cultured in cell lines and inactivated by exposure to formaldehyde and subsequently purified and stabilized for human applications. Early-phase clinical trials have been conducted on adult volunteers, and the vaccine was well tolerated without significant adverse reactions and induced robust immune responses with high titers of neutralizing antibodies until the 14th day after the second dose. However, the persistence of antibodies was not evaluated, and the investigation was discontinued [379].

Based on animal model studies (Table 5), the attenuated vaccine VLA1553 (IXCHIQ^®^, manufactured by the company Valvena, Saint-Herblain, France) was developed and clinical phase trials began. The phase I clinical trial evaluated the safety and immunogenicity of three increasing doses of the vaccine, including revaccination after 6 or 12 months of the last dose administered. A single dose induced strong neutralizing antibody production in most participants and immune responses remained elevated for months after vaccination (100% seroconversion rate). In addition, the vaccine was well tolerated, with only mild to moderate adverse effects, including participants who received a second dose [383].

Subsequently, phase III clinical trials were initiated with the aim of evaluating the safety and immunogenicity of VLA1553 up to 180 days after vaccination. The results showed that only one dose of the vaccine was required to induce a rapid and intense seroconversion response and the antibody titer remained elevated for at least six months, indicating that there was long-lasting immunity. As in other trials, the vaccine was well tolerated in healthy adults, presenting light adverse effects such as pain at the injection site, headache, fever and mild myalgia [384]. Other studies have been initiated to evaluate the persistence of antibodies and the safety of the CHIKV vaccine for up to 2 years [385], and to evaluate the efficacy in endemic areas and in adolescents (between 12 and 17 years of age) [386]. Based on these studies and the results obtained, the VLA1553 vaccine has been approved by the FDA (Silver Spring, MD, USA; 2023), the European Medicines Agency (EMA; European Union; 2024) [404] and by the Brazilian Agency equivalent to FDA and EMA, the “Agência Nacional de Vigilância Sanitária” (ANVISA; Brasília, Brazil; 2025) [405], in addition to being recommended by the Centers for Disease Control (CDC) for adults traveling to regions with known outbreaks of CHIKV [406].

## 7. Future Perspectives and Conclusions

Despite the high infection rate of CHIKV and significant expansion into new geographic areas, there are still no specific treatments. It is necessary to develop mechanisms to distinguish CHIKV infection from other arboviruses, in addition to understanding the impact of arbovirus co-infections on clinical manifestations [407].

In recent decades, significant advances have been made in understanding the pathogenesis of CHIKV, but studies that prioritize the mechanisms involved in the pain response are needed, especially in severe cases [407]. Painkillers and anti-inflammatories are commonly prescribed for patients along with medical monitoring. These approaches may not be enough, as they only alleviate symptoms such as fever, arthritis, and myalgia and do not prevent persistent inflammation or progression to chronic pain [293]. This results in an impact on the quality of life of those patients [3]. Currently, treatments for CHIKV infection are mostly symptomatic, without a direct focus on modulating the underlying disease mechanism [407]. The lack of data on the mechanistic details involved in CHIKV infection highlights the need to seek specific therapeutic targets to control the disease.

Antiviral therapy has been shown to be effective at inhibiting CHIKV replication, but due to the lack of knowledge about the pathogenesis of CHIKV and the dynamics of viral mutation, the license for antiviral therapies remains unavailable [407]. Recently, the use of vaccines has been explored, but many affected regions have limited infrastructure, making government collaboration and public policies crucial to reducing costs and expanding their use in vulnerable populations. The high incidence rates and the need for new methods to control the vector highlight the importance of ensuring proper funding and resources for CHIKV-endemic countries, especially in low-income ones [407,408]. In addition, further research to expand development of licensed vaccines to pediatric, immunocompromised, and pregnant populations is needed [408].

Significant efforts have been made in recent years, but further research on the following topics is still necessary: the interruption of the transmission chain by controlling vector density and distribution, the diagnosis of CHIKV infection, treatments aimed at controlling symptoms based on the viral mechanism, and the redirection of current treatments towards more effective treatments [407].

## Figures and Tables

**Table 1:** Different cellular immune responses triggered by Chikungunya virus infection.

Cell Type	Immune Response/Mediators Involved	Models	Consequences in CHIKV Infection	References
Dendritic Cell	Stimulates antiviral adaptive immune response. TNF-α, IL-12p70 and IFN-I cytokines release	C57BL/6 mice	Limits viral replication and dissemination	[224,228,259]
		Balb/c mice		
Neutrophil	Promote release of IL-1β, IFN-I, CXCL-1 and IL-6 cytokines and NET	C57BL/6 mice: WT, KO TLR7, KO IFNAR, KO IRF7, KO IRFNAR, KO IFN-β	Limits CHIKV replication and dissemination;	[232,233,234]
		Balb/c mice: WT, KO AnxA1	CHIKV neutralization	[233]
		C57BL/6 mice: WT, KO *Fpr2/3-*, KO IFN-β		
		C57BL/6 mice: WT, Arg1^Flox/Flox^ mice	Joint damage	[236]
			Arthritis	
Monocyte	Release IFN-α. Produces IL-6, TNF-α, and GM-CSF	C57BL/6 mice: WT, KO *Mavs*, KO *Irf3 e Irf7*, CCR2-DTR transgenic mice	Inhibits CHIKV replication	[116,221,240]
		Human serum		
		*Cynomolgus* macaques (*Macaca fascicularis*);	Joint pathology	[115,241]
		Human serum		
Macrophage	Produces IFN-I cytokines, reduces OPG. Increases RANKL and IL-6 expression. Induces osteoclastogenesis. NLPR3 activation and IL-1β release	C57BL/6 mice: KO IFN-α/Βr, KO *Nlpr3*, KO *Casp1*, KO *Aim2*, KO *Nlrc4*, KO *Asc*, KO *Il1r*	Limits viral replication	[119,244,260,261]
		Human serum		
		C57BL/6 mice: WT	Reduces viremia	[129]
			Articular and musculoskeletal damage	[129,221,244]
		Balb/c: CD1-mice		
NK cell	IFN-γ and TNF-α production. Cytotoxicity	C57BL/6 mice	Viral elimination	[221,241,251,256,257]
			Modulate the viral load	
		Human serum	Arthralgia	
			Clearance of infected cells	
CD4^+^ T lymphocyte	Release IFN-γ. Induces antibodies production. Joint pain and inflammation	C57BL/6 mice	Viral elimination. Joint inflammation	[217,222,256,262,263]
		Human serum		
CD8^+^ T lymphocyte	Immune response amplification. Cytotoxicity	C57BL/6 mice	Viral elimination. Infected host cells elimination. Joint inflammation	[217,263,264,265]
		Human serum		
Th1 lymphocyte	Release IFN-γ and TNF. Activates macrophages	Human serum	Viral elimination; Arthritis	[266,267]
Th2 lymphocyte	Release IL-4, IL-5 and IL-13. Stimulates B cells and antibodies production. Modulates Th1/Th17 responses	Human serum	Viral maintenance. Arthritis. Musculoskeletal pain. Chronification of the infection	[266,268]
Th17 lymphocyte	Produces IL-17, IL-6, IL-21, IL-22, IL-1β and TGF-β. Neutrophils recruitment and activation	DBA1/J mice	Reduces viral load. Inflammation and tissue damage. Joint and muscle pain. Arthritis	[263,266,269]
		Human serum		
T helper lymphocyte	Produces IL-10	Human serum	Inhibits immune response. Protection against CHIKV-induced pathology. Reduce viral load	[269,270]
B lymphocyte	Produces antibodies against CHIKV (IgM and IgG)	C57BL/6 mice: WT, KO Rag1	Limits viral replication and dissemination	[271,272,273]
		Human serum		

The type of cells involved in the immune response to CHIKV infection. Each cell type (DC, neutrophils, monocytes/macrophages, NK cells, T lymphocytes, and B lymphocytes) is indicated, along with their products/activity upon contact with CHIKV, and the subsequent effects of activating these cells on the infectious process.

**Table 2:** Humoral immune and antibodies response activated to Chikungunya Virus infection.

Response to CHIKV Infection	Chemical Mediator Class		Mediator	Models	Consequence in CHIKV Infection	References
Humoral immune response	Cytokines	Pro-inflammatory	IFN-α	In vitro: HeLa epithelial cell line; Vero WHO (ATCC)	Pain intensity. Control viremia. Reduce infection, viral load, polyarthritis, muscle pain, chronic joint pain	[4,116,217,221,249,268,287,288,289]
				In vivo: Human serum;		
			IFN-β	In vitro: Vero WHO (ATCC)	Fatigue. Reduce infection. Reduce viral replication	[268,287]
			IFN-ω	In vivo: Human serum	Reduce infection. Reduce viral replication	[268,287]
			IFN-γ	In vivo: Human serum	Low backache	[268,290]
			TNF-α	In vitro: *HEK293*/*T cells*	Headache. Persistent arthritis. Joint inflammation	[27,268,291,292]
			IL-1 β		Biomarkers. Disease severity. Induced fever. Persistent arthralgia. Joint inflammation	[27,268,289,291,292]
			IL-5		Persistent arthralgia. Joint inflammation	[27,291,292]
			IL-6	In vivo: Human serum	Skin rash. Persistent arthralgia. Biomarkers. Disease severity. Induce fever. Joint inflammation. Musculoskeletal pain. Osteoporosis. Viral load. Polyarthritis. Chronic joint pain	[4,27,217,221,248,249,268,288,289,291,292,293]
			IL-7		Persistent arthritis	[27,289,291,292]
			IL-8		Persistent arthralgia. Joint inflammation	[27,291,292]
			IL-12	In vivo: Human serum	Viral load	[249,288]
			GM-CSF	In vitro: *HEK293*/*T cells*	Persistent arthralgia. Joint inflammation	[27,291,292]
				In vivo: Human serum		
		Anti-inflammatory	IL-27	In vivo: Human	Act as reservoirs of CHIKV. Increase inflammatory process	[294]
			IL-13	In vitro: *HEK293*/*T cells*	Persistent arthralgia. Joint inflammation	[27,268,291,292]
				In vivo: Human serum		
			IL-4	In vivo: Human serum	Musculoskeletal pain	[268]
			IL-10		Myalgia. Reduce viral load. Resolution of infection. Musculoskeletal pain	[268,290,295,296]
			IL-17		Persistent arthralgia. Osteoporosis	[4,249,288,289,291,296]
	Chemokines		CCL2/MCP-1	In vivo: Female C57BL/6 mice; ***B***ALB/c mice; Human serum	Joint inflammation. Viral load. Viral clearance	[4,129,249,268,288,289,291,296,297,298]
			CXCL10/IP-10	In vivo: ***B***ALB/c mice; Human serum	Biomarker. Musculoskeletal pain. Viral load. Viral clearance. Polyarthritis. Chronic joint pain	[4,221,249,268,288,289,296,298]
			CXCL-9 (MIG)	In vivo: Human serum	Biomarker	[289,291,296]
			IL-8 (CXCL8)	In vivo: Human serum	Persistent arthritis. Joint inflammation	[289,291,296]
			RANTES (CCL5)	-	Biomarker. Disease severity. Persistent arthralgia. Joint inflammation	[289,291,292,296]
Antibodies			*IgM*	In vivo: Human serum	Biomarker	[296]
			*IgG*			
Others			CRP	In vivo: Human serum	Musculoskeletal pain	[268]
			GM-CSF		Arthralgia	[221,249]
			TRAF-6	In vivo: BALB/c mice	Viral clearance	[298]
			TLR3			
			IRF-1/3/7			
			TICAM-1			

The table summarizes the main cytokines (pro- and anti-inflammatory), chemokines, and antibody classes involved in CHIKV infection. The types of cytokines, chemokines, and antibodies are indicated, as well as the consequences of their production and release during CHIKV infection. Readers are referred to the references list for additional details.

**Table 3:** Main antiviral agents under investigation against Chikungunya virus.

Technology	Antiviral Agent	Model	Mechanism of Action	Reference
Entry Inhibitors	Chloroquine	In vitro: Vero Cells	Decreased viral infectivity, prevented endocytosis and/or endosomal acidification	[309]
		In vitro: HEK293T, BHK-21 and Vero cells	Neutralized endosomal pH and inhibited viral fusion	[181]
		In vivo: *Cynomolgus* monkeys	It increased viremia, severe lymphopenia, and slowed viral clearance, specific cellular response, and IgM production	[310]
		In vivo: Humans (Phase 3 Clinical Trial)	It did not reduce viral load, did not improve acute symptoms, and altered immune markers (CRP, IFN-α, IL-6, MCP-1)	[310] ClinicalTrials.gov: NCT00391313
		In vivo: Humans (Phase 3 Clinical Trial)	There were no significant differences, cytokine levels remained elevated	[311] ClinicalTrials.gov: NCT00391313
	EGCG	In vitro: HEK293T cells	Reduced infection and inhibited viral binding and entry to target cells	[312]
		In vitro: U2OS and BHK-21 cells	It inhibited viral RNA, progeny yield, and the cytopathic effect of CHIKV. Protected against viral entry, replication, and release	[313]
	Suramin	In vitro: U2OS, Sf21 and BHK-21 cells	It reduced plaque formation, viremia, protein expression, the release of viral particles and the yield of the virus. Interfered with cell binding and fusion	[314]
		In vitro: Vero E6 and BHK-210 cells	It inhibited RNA synthesis and replication. Interfered with adsorption, entry and fusion	[315]
		In vivo: C57BL/6 Mice	It decreased viral loads, improved acute lesions, restored cartilage integrity, and reduced the number of chondrocytes	[316]
		In vitro: HEK293T, MCF7 and Huh7 cells	It inhibited entry, adsorption, and fusion by blocking the binding of the virus to the cell surface. Reduces CHIKV infection	[317]
	Suramin-Based Compounds	In vitro: Vero E6 Cells	Inhibited CHIKV replication	[318]
	LQM3334	In vitro: Vero E6 Cells	Inhibited CHIKV replication	[319]
	Niclosamide	In vitro: BHK-21 and Sf21 cells	Limits viral entry, inhibits virus release and transmission	[320]
	Nitazoxanide			
	Sulfonylamidine 1	In vitro: Hek293T/17 Cells	Inhibited viral entry and production	[321]
	Flavaglina FL3			
	Flavaglina FL23			
	Imipramine	In vitro: HFF1 cells	Inhibited viral replication	[322]
RNA Interference	CK1 (siRNA-nsP3) CK2 (siRNA-E1)	In vitro: Vero Cells	Reduced viral titers and genomic RNA of the virus	[323]
	shRNA-E1 shRNA-NsP1	In vitro: HeLa, BHK-21, and RD cells	Suppressed viral replication	[324]
		In vivo: C57BL/6 Mice	Protected against infection	
	amiRNA-Capsid amiRNA-nsP1 ami-RNA-nsP2	In vitro: Vero Cells	Inhibited replication and infection	[325]
	HSP-90	In vitro: BHK-21 and HEK239T cells	Reduced viral replication	[326]
nsP1 inhibitors	Lobaric Acid	In vitro: BHK-21 and Huh7 cells	Inhibited GTP binding to nsP1 active site, guanylylation, and viral growth	[327]
	([1,2,3]triazole [4,5-d]pyrimidin-7(6H)-ones)	In vitro: Vero Cells	Modulated nsP1 capping activity and stopped viral cycle	[328]
	Derivatives of ([1,2,3]triazole [4,5-d]pyrimidin-7(6H)-ones)	In vitro: Vero Cells	Inhibited nsP1 guanylylation activity and viral replication	[329]
			Inhibited viral replication	[330]
	Tetrahydropyranyl	In vitro: HFF1 cells	Antiviral activity	[331]
	N-t-butylpiperidine carboxamide			
	Analogues of 2-(4-(phenylsulfonyl)piperazine-1-yl)pyrimidine	-	Antiviral activity	[332]
	6′-β-fluoro-homoarymycin	In vitro: Vero E6 Cells	Inhibited viral entry and replication, decreases infectivity	[333,334]
	6′-fluoro-homoneplanocin A			[333]
	5-Iodotubercidine	In vitro: Vero Cells	Antiviral activity	[335]
nsP2 inhibitors	CMPD222	Molecular docking; Molecular dynamics simulations	Inhibition of the nsP2 protein	[336]
	Plant Bioactives	Molecular docking	Inhibition of nsP2 and E1 protein	[337]
	Ribostamycin Sulfate	Molecular docking; Molecular dynamics simulations	Inhibition of the nsP2 protein	[338]
	E-64			
	Compound 8	Molecular docking In vitro: BHK-21 Cells	Inhibition of the nsP2 protein. It inhibited the production of viral RNA, and reduced the production and release of new virions	[339]
	Compound 7			
	Compound 9			
	MBZM-N-IBT	In vitro: Vero Cells	It decreased the formation of viral particles and the synthesis of viral RNA. It reduced the levels of the proteins nsP2 and E2. Inhibited infection in early and late stages	[340]
		In vitro: RAW Cells 264.7	It inhibited infection and viral proteins, interfering in the early stages. Decreased inflammatory responses through downregulation of MAPKs, NF-κB, COX-2, and cytokines	[341]
		In vivo: C57BL/6 Mice	Decreased infection and inflammation. Increased survival rate	
		Ex vivo: hPBMCs cells	Reduced infection	
	Arylalkylidene derivatives of 1,3-thiazolidin-4-one (1–20)	In vitro: Vero Cells	Inhibited infection	[342]
	3a, 4b and 5 d (peptidomimetics)	Molecular docking In vitro: Vero Cells	Inhibited viral replication	[343]
	Pep-I and Pep-II (peptidomimetics)	In vitro: BHK-21 Cells	Inhibited viral replication	[344]
	ID1452-2	In vitro: HEK-293T Cells	Inhibited viral replication and partially blocked nsP2 activity	[345]
	Telmisartan	In vitro: Vero Cells	Suppressed viral spread	[346]
	Novobiocin			
	Nelfinavir	In vitro: Vero Cells	Antiviral activity	[347]
nsP3 inhibitors	2-oxo-5,6-benzopyrimidine-4-carboxylic acid	In vitro: ATCC Cells	Antiviral activity	[348]
	Harringtonin	In vitro: BHK-21, C6/36 and HSMM cells	Inhibited infection and the early stage of the replication cycle. Blocked the expression of the nsP3 protein	[349]
nsP4 inhibitors	β-D-N4-Hydroxycytidine	In vitro: BHK-21 and Huh-7 cells	Inhibited viral replication	[350]
	6-azauridine	In vitro: Vero Cells	Antiviral activity	[351]
	Sofosbuvir	In vitro: Vero and Huh-7 cells	Inhibited viral replication	[352]
		In vivo: Swiss Mice	Prevented edema and protected against mortality	
	Ribavirin	In vitro: Vero, A549 and Huh-7 cells	Increased antiviral activity in Huh-7 cells Reduced viral load	[353]
	Favipiravir			
	IFN-α		Increased antiviral activity in A549 cells. Reduced viral load	
	Ribavirin + Doxycycline	In vitro: Vero Cells	Inhibited viral replication	[354]
		In vivo: ICR mice	Attenuated infectivity	
	Ribavirin + IFN-α	In vitro: Vero Cells	Antiviral activity	[355]
	Favipiravir	In vitro: Vero Cells	Inhibited viral replication	[330]
		In vivo: AG129 Mice	Reduced infection	
		In vivo: C57BL/6 Mice	Inhibited systemic viral spread	[356]
	Defluorinated Analog of Favipiravir (T-1105)	In vitro: Vero Cells	Inhibited viral replication	[330]
	Mycophenolic Acid	In vitro: Vero Cells	It reduced viral titers and inhibited virus-induced apoptosis. Blocked the formation of viral progeny	[357]
	Compound A (Benzimidazole)	In vitro: Vero, BHK-21 and HEK-293T cells	Antiviral activity	[358]
	5b, 5c and 5d (Quinolone-N-acyl-hydrazone hybrids)	In vitro: Vero Cells	Inhibited viral RNA production	[359]
Protein C Inhibitors	Picolini Acid	In vitro: Vero Cells	Reduced RNA production and viral titer	[360]
	Mandelic Acid	Molecular docking; Surface plasmonic resonance; Fluorescence Spectroscopy	Binds to protein C	[361]
	Ethyl-3-Aminobenzoate			
	P1,P4-Di(adenosine-5′) tetraphosphate	-	Inhibited the autoproteolytic activity of protein C	[362]
	Eptifibatide Acetate			
	Paromomycin Sulfate			
6K Protein Inhibitors	Amantadine	In vitro: Vero Cells	It reduced viral particle production, replication and viral titer. It disturbed the maturation and assembly of the vital envelope	[363]

This table summarizes the main antivirals in development against the Chikungunya virus, highlighting the technology employed, the agents tested, the experimental models used and their mechanisms of action.

**Table 4:** Main studies under development with polyclonal and monoclonal antibodies against Chikungunya virus.

Technology	Antibody	Model	Mechanism of Action	Reference
Polyclonal Antibodies	CHIKVIg	In vivo: IFN-α/βr^−/−^129s/v and C57BL/6 mice	It neutralized the CHIKV, prevented disease, inhibited viremia and clinical signs. In neonates, it reduced mortality, viral load and symptoms	[374]
	Anti-CHIKV Hyperimmune Immunoglobulin	In vivo: Human (Phase I and II Clinical Trials)	No results so far	ClinicalTrials.gov: NCT02230163
Monoclonal Antibodies	Anti-MXRA8	In vitro: Vero cells, NIH-3T3, MEFs, HEK293T, A549, HeLa, MRC-5, HFF-1, Hs633T, Huh-7, RPE, JEG3, U2OS, HT1080, Raji and K562	Blocked CHIKV infection	[195]
		In vivo: C57BL/6J mice	Reduced viral load and clinical signs of infection	
	DC2.112 and DC2.315	In vitro: Vero cells and human PBMCs	Low neutralization. Inhibited viral release and recruited myeloid cells to promote phagocytosis of infected cells	[375]
		In vivo: C57BL/6J and FcγR^−/−^C57BL/6 mice	Protected against infection	
	Anti-E1	In vitro: Vero Cells	Inhibited the release of new virions	[376]

This table summarizes the main polyclonal and monoclonal antibodies under development against the Chikungunya virus, highlighting the technology employed, the antibodies tested, the experimental models used and their mechanisms of action.

**Table 5:** Main vaccines in development against Chikungunya virus.

Technology	Vaccine	Model	Mechanism of Action	Reference
Inactivated Vaccines	BBV87	In vivo: Mice	Induced high titers of neutralizing antibodies and protective immune response	[378]
		In vivo: Human (Phase I and II Clinical Trials)	It induced 100% seroconversion and high titers of long-lasting neutralizing antibodies. It was well tolerated	
		In vivo: Human (Phase II and III Clinical Trials)	No results so far	ClinicalTrials.gov: NCT04566484
	Inactivated by Formalin	In vivo: Mice; *Rhesus* monkeys	Induced high titers of neutralizing antibodies and protected the animals	[379]
		In vivo: Human (Early phase clinical trials)	It induced high titers of neutralizing antibodies and elicited robust immune responses. It was well tolerated, but studies were discontinued	
		In vivo: Mice	Induced high titers of neutralizing antibodies and IgG	[380]
		In vitro: Spleen cells from vaccinated animals	It produced IFN-γ, IL-2 and IL-4. Antibodies blocked the infection	
	HydroVax-CHIKV	In vivo: BALB/c ByJ mice	It induced elevated neutralizing responses and protected animals against viremia, arthritic disease, and lethal infection	[381]
Live Attenuated Vaccines	Δ5nsP3	In vivo: C57BL/6 Mice	Induced neutralizing antibody production and T cell responses. Protected from viremia and joint swelling	[382]
	Δ6K			
	VLA1553 (IXCHIQ)^®^	In vivo: Human (Phase I Clinical Trials)	It induced strong neutralizing antibody production and long-lasting immune responses. It was well tolerated	[383]
		In vivo: Human (Phase III Clinical Trials)	It induced rapid and intense seroconversion and high titers of long-lasting neutralizing antibodies. It was well tolerated	[384]
		In vivo: Human (Phase III Clinical Trials)	No results so far	[385,386]
	ΔC-CHIKV	In vivo: C57BL/6 Mice	Induced protective and complete immune responses	[187]
	VEEV-ΔC-CHIKV	In vivo: C57BL/6 and IFNAR^−/−^ mice	Protected against infection and prevented viremia	[387]
	Δ5nsP3	In vivo: *Cynomolgus* monkeys; Macaca *fascicularis*	Induced robust neutralizing antibody responses. It protected against viremia and the clinical signs of the disease. It was safe	[388]
	DREP-E			
	DREP-E prime + MVA-CE			
	CHIKV-NoLS	In vivo: C57BL/6 Mice	Protected against infection, viremia, and clinical signs of disease	[389]
	CHIKV-R532H	In vivo: C57BL/6 Mice	Protected against infection, viremia, and clinical signs of disease	[390]
	Stop CHIKV	In vitro: Vero, 293T, and BHK-21 cells	Antiviral activity	[391]
		In vivo: C57BL/6 Mice	It inhibited viremia, viral spread and replication. Induced neutralizing antibodies	
	SuperStop CHIKV	In vitro: Vero, 293T, and BHK-21 cells	Antiviral activity	
		In vivo: C57BL/6 Mice	It inhibited viremia, viral spread and replication. Induced neutralizing antibodies	
	CHIKV HR	In vivo: C57BL/6J mice	It protected from the development of disease and viral persistence. Stopped viral replication	[392]
Chimeric and Recombinant Vaccines	TC-83 + CHIKV	In vivo: C57BL/6J and Swiss mice	It induced high titers of neutralizing antibodies. Protected against viremia and clinical signs	[393]
	EEEV + CHIKV			
	Sindbis + CHIKV			
	VEEV + CHIKV	In vivo: IFN-α/βr^−/−^ mice (A129)	It induced high titers of neutralizing antibodies. Protected against infection	[394]
	ISV-CHIKV	In vivo: IFN-α/βr^−/−^ mice (A129)	It induced high titers of neutralizing antibodies. Reduced viremia	[395]
	Ad5 CHIKV	In vivo: CD-1 and C57BL/6 mice	Protected animals from viremia and clinical signs	[396]
DNA vaccines	pCHIKV-E1	In vivo: BALB/c mice	Induced anti-hemagglutination antibodies	[397]
	pCHIKV-Capsid		It did not generate antibodies	
	PMCE321		It induced high titers of neutralizing antibodies and hemagglutination inhibitors. Activated T-cell responses and protected against infection	
	PEnv + pnsP2	In vivo: C57BL/6 Mice	It delayed the onset of symptoms, reduced morbidity. Induced neutralizing antibodies	[398]
	DNA Plasmid + Neutralizing Antibody	In vivo: BALB/c mice	Induced high levels of circulating antibodies and protected against infection	[399]
RNA vaccines	LNP-CHIKV	In vivo: C57BL/6 Mice	Induced high titers of neutralizing antibodies and T cell responses. Produced INF-γ, TNF-α, and IL-2	[400]
	LNP-encapsulated CHIKV-NoLS RNA	In vivo: C57BL/6 and AG129 mice	Protected after additional reinforcements	[401]
	iRNA CHIKV	In vivo: C57BL/6 Mice	It induced binding and neutralizing antibody responses. Protected against viremia	[402]
VLP	Sf21 de Spodoptera frugiperda + CHIKV	In vivo: C57BL/6 Mice	It induced high titers of neutralizing antibodies. Protected against viremia	[168]
	Pichia pastoris + CHIKV	In vivo: BALB/c mice	It induced high titers of IgG antibodies and produced TNF-α, IL-10, IL-2, IL-4 and IFN-γ. Protected against infection	[403]

This table summarizes the main vaccines under development against the Chikungunya virus, highlighting the technology employed, the vaccines tested, the experimental models used and their mechanisms of action.

## Data Availability

No new data were created or analyzed in this study. Data sharing is not applicable to this article.

## Abbreviations

The following abbreviations are used in this manuscript:

CHIKV	Chikungunya Virus
Ae	Aedes
CHIKF	Chikungunya Fever
USA	United States of America
ECSA	East Central South African
IOL	Indian Ocean Lineage
Wb	*Wolbachia*
TEP	Thioester-Containing Proteins
GNBPB1	Glycosaminoglycan-Binding Protein B1
PZ1A	Serine Protease Z1A
NHP	Non-Human Primate
TRPV1	Transient Receptor Potential Vanilloid 1
IFN	Interferon
IFN-I	Type I Interferon
DC	Dendritic Cells
IL	Interleukin
IL1Ra	Interleukin-1 Receptor Antagonist
MCP-1	Monocyte Chemotactic Protein-1
VEGF	Vascular Endothelial Growth Factor
RNA	Ribonucleic Acid
SINV	Sindbis Virus
EEEV	Eastern Equine Encephalitis Virus
Poli(A)	Polyadenylated
ORF	Open Reading Frames
nsP	Non-Structural Proteins
RTPase	RNA triphosphatase
ADP	Adenosine Diphosphate
CP	Capsid Protein
TF	Transferase
Mxra8	Matrix Remodeling-Associated Protein 8
ASR	Acid-Sensitive Region
PHB1	Prohibitin-1
GAG	Glycosaminoglycans
PtdSer	Phosphatidylserine
TIM-1	T-cell Immunoglobulin and Mucin Domain
CME	Clathrin-Mediated Endocytosis
AP-2	Adaptor Protein-2
Eps15	Epidermal Growth Factor Receptor Substrate 15
GTPase	Guanosine Triphosphatase
PRR	Pattern Recognition Receptors
NLR	NOD-Like Receptor
TLR	Toll-Like Receptor
NF-κB	Nuclear Factor Kappa B
IRF	Interferon Regulatory Factor
CCL	C-C Motif Chemokine Ligand
NK	Natural Killer
Ig	Immunoglobulin
TNF	Tumor Necrosis Factor
KO	Knockout
MyD88	Myeloid Differentiation Primary Response Gene 88
DCIR	Dendritic Cell Immunoreceptor
CXCL	Chemokine (CXC Motif) Ligand
TLR7	Toll like receptor 7
ROS	Reactive Oxygen Species
NET	Neutrophil Extracellular Traps
IFNAR	IFN Alpha/Beta Receptor
MPO	Myeloperoxidase
GM-CSF	Granulocyte Macrophage Colony Stimulating Factor
RANKL	Receptor Activator of Nuclear Factor Kappa-B Ligand
OPG	Osteoprotegerin
KIR	Killer Cell Immunoglobulin-Like Receptor
HLA	Human Leukocyte Antigen
NCR	Natural Cytotoxicity Receptor
Treg	Regulatory T
Th	Helper T Cells
IgM	Immunoglobulin M
IgG	Immunoglobulin G
DENV	Dengue Virus
ZIKV	Zika Virus
RT-PCR	Reverse Transcription Polymerase Chain Reaction
ELISA	Enzyme-Linked Immunosorbent Assays
NSAID	Nonsteroidal Anti-Inflammatory Drugs
RBV	Ribavirin
DMARD	Disease-Modifying Antirheumatic Drugs
MTX	Methotrexate
EGCG	Epigallocatechin-3-Gallate
FDA	Food and Drug Administration
AIDS	Acquired Immunodeficiency Syndrome
RNAi	Interference RNA
mRNA	Messenger Ribonucleic Acid
shRNA	Small Loop Interfering RNA
amiRNA	Artificial MicroRNA
MTase	Methyltransferase
GTase	Guanylyltransferase
GTP	Guanosine Triphosphate
SAR	Structure-Activity Studies
FHA	6′-β-fluorohomoaryromycin
FHNA	6′-fluoro-homoplanocin A
5-IT	5-iodotububercidin
NTPase	Nucleoside-Triphosphatase
RNAse	RNA Triphosphatase
MBZM-N-IBT	1-[(2-methylbenzimidazole-1-yl)methyl]-2-oxo-indolin-3-ylidene]amino]thiourea
PFU	Plaque-Forming Unit
RdRp	RNA-dependent RNA polymerase
NA	Nucleoside Analogues
NHC	β-d-N4-hydroxycytidine
IMPDH	Inosine Monophosphate Dehydrogenase
FAV	Favipiravir
MPA	Mycophenolic Acid
PARP	Poly(ADP-Ribose) Polymerase
Bcl-2	B-Cell Lymphoma 2
PCA	Picolinic Acid
ITC	Isothermal Titration Calorimetry
SPR	Surface Plasmon Resonance
MDA	Mandelic Acid
EAB	Ethyl 3-Aminobenzoate (EAB)
AP4	P1,P4-Di(adenosine-5′)tetraphosphate
EAC	Eptifibatide Acetate
PSU	Paromomycin Sulfate
pAb	Polyclonal Antibodies
mAb	Monoclonal Antibodies
PRNT	Plaque Reduction Neutralization Tests
CRISPR	Clustered Regularly Interspaced Short Palindromic Repeats
LAV	Live Attenuated Vaccines
BPL	β-Propiolactone
EMA	European Medicines Agency
ANVISA	Agência Nacional de Vigilância Sanitária
CDC	Centers for Disease Control
